# Comparative evaluation of two autotransfusion devices in a 72h survival swine model of surgically induced controlled splenic bleeding

**DOI:** 10.1371/journal.pone.0322568

**Published:** 2025-05-05

**Authors:** Axelle Castelli, Chloé Libaud, Benoît Decouture, Marine Bruneau, Mallorie Depond, Patricia Forest-Villegas, Olivier Gauthier, Audrey Lafragette, Gwenola Touzot-Jourde

**Affiliations:** 1 CRIP, Center for Research and Preclinical Investigation, Oniris Nantes Atlantic College of Veterinary Medicine, Food Science and Engineering, Nantes, France; 2 i-SEP, Nantes, France; 3 Regenerative Medicine and Skeleton, INSERM, ONIRIS, Nantes University, UMR 1229- RMeS, Nantes, France; 4 Ecole Nationale Vétérinaire d’Alfort, ACAP3, Goustranville, France; 5 Ecole Nationale Vétérinaire d’Alfort, Maisons-Alfort, France; Kamuzu University of Health Sciences, MALAWI

## Abstract

Autotransfusion is a key strategy in hemorrhagic surgical procedures, reducing risks like disease transmission and immunosuppression due to allogenic transfusion. While conventional devices efficiently process red blood cells by centrifugation, they don’t address complications requiring additional platelet transfusions. The innovative same™ device (i-SEP, France), utilizing hollow-fiber filtration, preserves both red blood cells and platelets without damaging cell integrity. This study designed as a prospective randomized controlled trial compared cell concentration and washout performances of two autotransfusion devices, a preclinical version of same™, the conventional centrifugation-based Xtra® (LivaNova, UK); and effects of retransfusion versus no transfusion in 21 Yucatan minipigs submitted to a surgically induced controlled splenic bleeding. Animals were divided into three groups (no-transfusion *control* group, *same* group and *xtra* group) and monitored postoperatively for 72 hours during which serial animal blood samples were collected for hematologic, biochemical and coagulation analyses and tests. Both autotransfusion devices showed high performances in red blood cell yields and concentrations, with a significant superiority of same™ device to preserve platelets. Animals from *same* and *xtra* groups retrieved similar rheological profiles and maintained a normal hematocrit compared to no-transfusion control animals. Coagulation profiles stayed within normal range in all groups. No adverse event on animals nor post-mortem sign of thrombosis were identified on autotransfused animals. The same™ device that can therefore be considered as an interesting alternative to conventional centrifugation-based devices. Further experiments are needed to provide evidence of platelets autotransfusion benefits in massive hemorrhagic procedures.

## Introduction

Autotransfusion is an increasingly-used attractive patient blood management strategy. Red blood cell (RBC) autotransfusion allows to reduce the need for allogenic blood transfusions (donor) in numerous surgical procedures (cardiac, orthopedic, visceral, vascular, obstetric) [1–3]. Benefits of autologous transfusion lay in the absence of infectious disease transmission, transfusion reactions and immunosuppression [4–8], while not carrying allogenic transfusion limits like is donor’s availability and high processing costs [9–12]. Autotransfusion is an important pillar in the patient blood management strategy and the use of an autotransfusion device is recommended by international guidelines during procedures in which blood loss is anticipated [13,14]. Conventional autotransfusion devices process the collected blood mainly by centrifugation and provide a red blood cell concentrate for patient transfusion while removing platelets and coagulation factors with the potential of leading to dilutional coagulopathy if large blood volumes are treated and reinfused to the patient [15–17]. Resulting thrombocytopenia increases the need for platelet transfusion that is associated with an increase in postoperative complications including infections and increased length of hospital stay [14,18–21]. These disadvantages point out the need for a device able to simultaneously wash and concentrate RBC and platelets while preserving cell integrity.

The Xtra^®^ device (LivaNova, London, UK) is one of the gold-standard cell salvage systems used in perioperative conditions for autologous blood recovery [22–24]. This device collects blood, washes and concentrates mainly red blood cells (RBC) by centrifugation within the intra-operative period [25].

The new autotransfusion medical device based on a hollow-fiber filtration technology (same™ by i-SEP, Nantes, France) has been shown in an *in vitro* study to efficiently wash and concentrate RBC as well as platelets without significant impact on cell integrity and function [26,27]. Indeed, RBC and platelet functionalities were preserved while leukocytes did not show abnormal activation nor cell death. The same™ device has then been evaluated in an *in vivo* study through a swine model reproducing two clinicals conditions of surgically induced blood loss: cardiac and visceral blood loss [27]. Pigs reinfused with same™ device treated blood had minimal variations in their whole blood count, hematocrit, hemoglobin and red blood cell concentrations during the surgical procedure and the 72 h postoperative follow-up time. The reinfusion did not lead to any postoperative adverse events. No hypo- nor hyper-coagulable state was evidenced and postmortem histological examination did not reveal any thrombotic lesion [27]. The study collecting *in vivo* parameters confirmed results obtained in the previous *in vitro* study by Mansour et al. [26].

The present study aimed at prospectively comparing two autotransfusion devices: the filtration-based same™ device and the centrifugation-based Xtra^®^ device in an *in vivo* porcine model of surgically-induced abdominal controlled hemorrhage, with a targeted blood-loss reaching 15% of the animal total blood volume [28], with a 72-hour survival. The aims were to compare device performances for blood treatment and document autotransfusion effects on animals with the blood treated by each device as well as animal recovery against a no-transfusion control group. This study also documents effects on hematology, coagulation and animal recovery including post-transfusion reactions and complications, comparatively to a no-transfusion control group.

## Materials and methods

This preclinical study was conducted in accordance with the regulations governing good laboratory practice: The Organization for Economic Co-Operation and Development Principles of Good Laboratory Practice [29] and the United States Food and Drug Administration Good Laboratory Practice for Non-Clinical Laboratory Studies (FDA-CFR 21 Part 58) [30]. The animal study protocol was run according to the European Community Guidelines for the care and use of laboratory animals (2010/63/UE) following approval by the Pays de La Loire ethical committee for animal experimentation and authorization by the French Ministry of Higher Education, Research and Innovation (Apafis number #11079-2017071317574824v1). All procedures were performed in an animal approved facility (Oniris Experimental Surgical Plateform, accreditation number C-44-271 by Loire Atlantique prefectoral order N° 2014-DDPP-132) and under guidance of the facility animal welfare body.

### Study design, autotransfusion devices and treatment group allocation

The study was designed as a prospective randomized controlled trial using a previously described animal model of visceral blood loss by controlled splenic bleeding induced surgically in minipigs [27]. Animals were randomly allocated to 3 treatment groups: 2 autotransfused groups with either device: same™ (2019 preclinical version, i-SEP, France) – named *same* group for the *in vivo* part of the study, or Xtra^®^ (LivaNova, UK) – named *xtra* group for the *in vivo* part of the study, and a group without transfusion after hemorrhage – named *control* group in *in vivo* study, with inclusion of 7 animals per group. The number of animals required was determined by a power calculation detailed in the supporting information, S1 File. The shed blood of control animals was randomly allocated to be treated either by the preclinical same™ device or the commercialized Xtra^®^ device in order to increase the total number of treated bloods per device from 7 to 10 for performance evaluation. The final product obtained from control animal blood was not reinfused and therefore discarded.

### Blood collection and autotransfusion treatment programs evaluation

The cell salvage treatment was performed in accordance with the manufacturer’s instructions for use and the recommended settings for processing and washing. Using the Xtra^®^ device, the blood was processed in the kit X/125 mL size bowl using the device’s built-in Intraoperative Standard Program (Pstd protocol) in Automatic Mode. Using the same™ device, the blood was processed in ST0300FR set with Standard mode. Detailed process of same™ device is described in previous published *in vitro* and *in vivo* studies [26,27]. Each autotransfusion devices were used to collect about 850-1000mL of diluted shed blood by animal from the surgical field, allowing to proceed to at least two consecutive treatment cycles, named cycle 1 and cycle 2. Blood samples were collected for analysis before and after each treatment cycle, in the collecting reservoir (TR) and in the transfusion bag (TB) respectively.

Qualitative and quantitative parameters were used to evaluate and compare the performances of the autotransfusion systems in washing and concentrating collected blood (RBC numeration and yield, hematocrit, hemoglobin concentration, heparin, free hemoglobin and other plasma components washout) following AABB recommendations and conventional practices [26,27], along with complementary parameters as platelet concentration and yield. Performances were calculated by formulas detailed in the supporting information, S1 File.

**Table 1:** Expected performances for criteria.

Parameters	Criteria	Ref.
*Quality of the blood to be reinfused – unwanted substances clearance*		
Anticoagulant (heparin) concentration (IU/mL)	≤0.5	a
Anticoagulant clearance (%)	>90%	a
Hemolysis rate (%)	≤0.8%	b
Free hemoglobin washout (%)	90%	a
Other plasma components washout (%)		b
*Quantification of blood elements to transfuse and device performances*		
Red blood cells yield (%)	> 80%	b
Final Hematocrit (%)	45% < Ht > 65%	a
Platelet yield (%)	NA	NA
Platelets Concentration (10^6^/µL)	NA	NA

a. Based on AABB recommendations [31].

b. Based on literature and conventional practices [32–34].

NA, not applicable.

### Animals and surgical model of abdominal controlled blood loss

**Fig 1:**
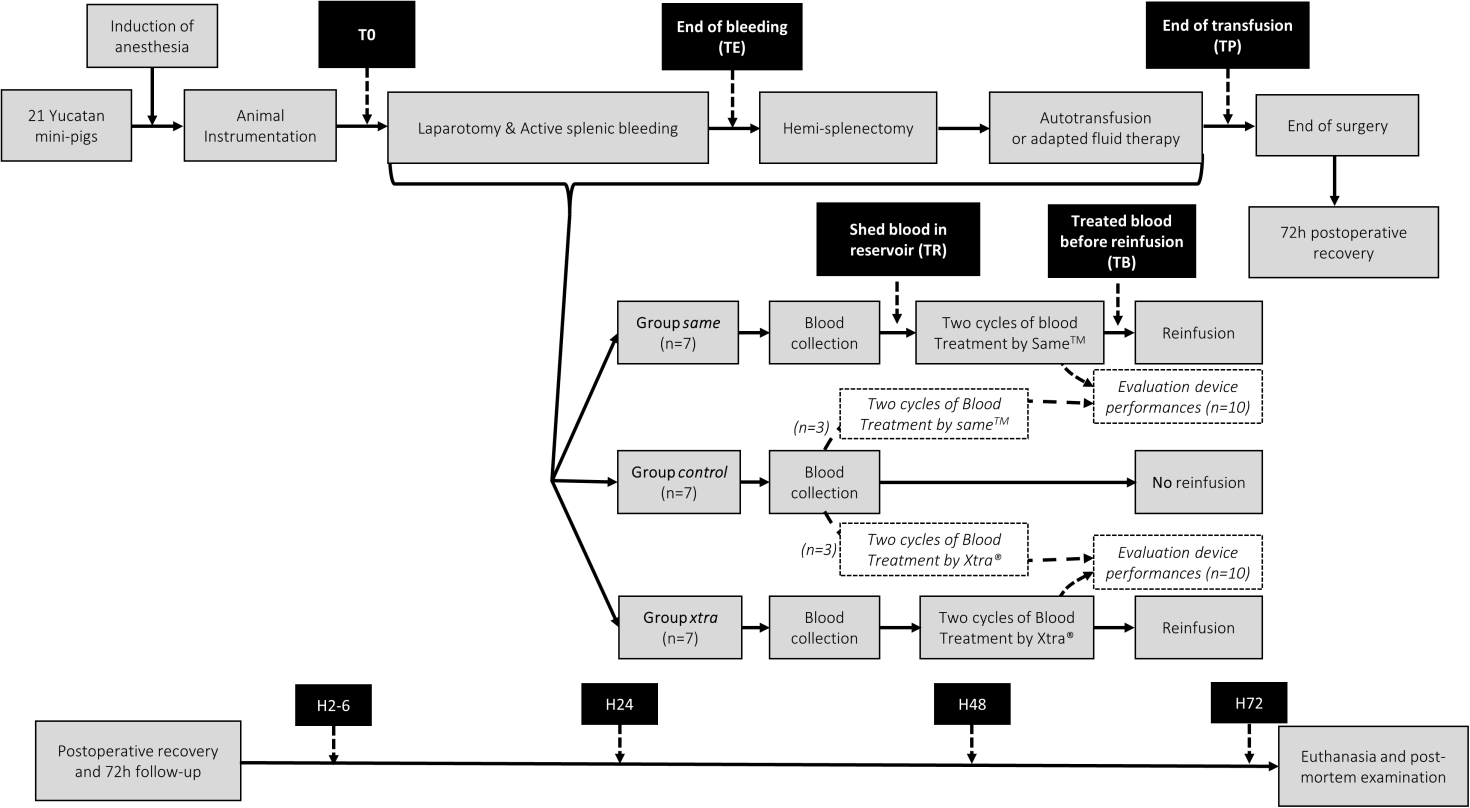
Timeline synopsis of surgery and blood samples management. Times of sampling were before surgery once the central venous catheter was in place (T0), at the end of bleeding (TE), at the end of the transfusion (TP), then postoperatively between 2 and 6 hours (H2), at 24 (H24), 48 (H48) and 72 hours post-transfusion (H72). Additional blood samples were obtained from the BCR (TR) and from the transfusion bag (TB) before the reinfusion started.

Twenty-one 14- to 22-month-old female, weighing 41.6 to 60.5 kg Yucatan minipigs were included in this study. Each animal was individually identified by an ear tag (since a few days after birth) to guarantee traceability. Detailed animal care, anesthesia and analgesia protocol as well as surgical model of abdominal controlled blood loss have been described in [27] and in the supporting information, S1 File. Briefly, after a week of acclimation in an enriched environment, animals were immobilized by an intramuscular injection. During anesthesia maintained with a balanced technique under controlled ventilation, arterial and central venous accesses were gained before surgically-induced controlled bleeding and autotransfusion. To counteract known porcine hypercoagulability [35–37] and to facilitate blood loss and collection, a low dose of unfractionated heparin (Heparin sodium 5 000 IU/mL, 25 IU/kg IV) was administered just before initiating the splenic injury. This dosage has been used in previous studies of porcine intra-abdominal hemorrhage and has shown to bring the porcine coagulation profile within values found in humans [27,38,39]. ACT measurements (Activated Clotting Time, via Medtronic ACT II Coagulation Timer) were done before heparin administration, repeated during the blood collection time (target ACT 90–130 seconds) and just before transfusion to ensure a return to baseline ACT value. Following a midline laparotomy, multiple lesions were created into the splenic parenchyma using digitoclastic technique and an additional lesion was done by severing the gastroepiploic vein. Spontaneous bleeding was let to drip into the abdominal cavity and then aspirated through the suction line (depression kept under 200 mbar to minimize hemolysis) [39] into the autotransfusion device blood collection reservoir (BCR). The target volume for blood loss was set to 15% of the estimated animal blood volume (61–68 ml/kg, calculus method in S1 File) [40] in order to reach the first stage of hemorrhagic shock inducing substantial hemodynamic compromises [28,41]. Taking into consideration that the spleen is responsible for platelet clearance from blood circulation and that maintaining splenic function postoperatively is necessary to assess physiological response to autotransfusion [42], surgically-induced splenic bleeding was controlled by performing a hemi-splenectomy using an endoscopic surgical stapler with 60 mm-long cartridges (Echelon Flex™ Endopath^®^, Ethicon Endosurgery, Johnson and Johnson). Left gastroepiploic vessels were then coagulated using a tissue fusion device (Atlas Ligasure™, Covidien, Medtronic). After the end of surgery and autotransfusion, the animals were allowed to recover and were euthanized at the end of a 72h follow-up period after transfusion completion. A timeline synopsis synthetizes the schedule of the operating theater and the blood samples management (Fig 1). Anesthesia depth and blood pressure support (intravenous fluid replacement and catecholamines) were adjusted to the animal response to acute blood loss to maintain a mean blood pressure above 60 mmHg while waiting for autotransfusion and if retransfusion did not stabilize sufficiently hemodynamic parameters. Postoperative care comprising analgesia and supportive therapy was adapted to each individual recovery during a 72-hour follow-up period post-hemorrhage until euthanasia. Animal welfare as well as anesthesia procedure, postoperative care and postoperative follow-up are detailed in S1 File. Euthanasia and post-mortem examination were geared to assess thrombogenic risk as described in the supporting information, S1 File.

### Laboratory testing

Blood samples were collected from before surgery to the end of the 72-hour follow up to monitor possible variations in hematologic and coagulation parameters. Times of sampling are described in Fig 1. Total blood volume collected was refined by establishing the minimal volume needed for laboratory testing ahead of the experiment. All blood samplings were performed by gentle aspiration through the central venous catheter which allowed stress-free repetitive procedures on the minipigs postoperatively. Additional blood samples were obtained from the BCR (TR) and from the transfusion bag (TB) before the reinfusion started. Blood analyses consisted of complete blood count (CBC) including RBC, WBC and platelet count, hematocrit and total hemoglobin (Procyte Dx Hematology Analyzer, IDEXX, Hoofddorp, The Netherlands), plasma free hemoglobin concentration measurement for the calculation of the hemolysis washout (Plasma/Low Hb photometer, HemoCue AB, Sweden), biochemical assays: sodium, chloride, potassium (VetLyte, IDEXX, The Netherlands); calcium, phosphate, glucose, lactate deshydrogenase, total protein, albumin, triglycerides, lactate (RX Daytona VetTest, IDEXX, The Netherlands); non-esterified fatty acids (NEFA FS, Diasys), TNF-alpha (Porcine TNF-alpha ELISA kit, R&D Systems), pig-map (Pig MAP (major acute phase protein) ELISA kit, FineTest), haptoglobin (haptoglobin assay, Tridelta PHASE), c-reactive protein (porcine CRP assay kit, Tridelta Phase), D-Dimers (D-Dimer ELISA kit, Technozym), plasma heparin concentration measurement through anti-Xa activity assay (Heparin standard-HNF, HemosIL Liquid anti-Xa, ACLTOP, IL, Werfen, France). Coagulation status was assessed by thromboelastometry with ROTEM non heparin sensitive EXTEM test (ROTEM delta, Werfen, France). Analyzed parameters were clotting time (CT; s), maximum clotting firmness (MCF; mm), AUC (a.u.), α angle (°) and clot lysis at 30 minutes (Ly30; %). Classical coagulation tests (fibrinogen, prothrombin time and activated partial thromboplastin time) were also performed (Start 4^®^, STAGO, France).

### Statistical analysis

Statistical analyses of continuous variables were performed with Multiple Mann-Whitney test and demographic data was compared between groups with a Kruskal-Wallis for non-parametric data. All the data are presented as median (min-max). All statistical tests were two-tailed, a ROUT (Prism adapted Dixon’s Q) test was performed with 1% FDR to exclude outlier values. A p-value of less than 0.05 was considered statistically significant and represented by stars in tables and figures where * is ≤0.05, ** is ≤0.01, *** ≤0.005, **** ≤0.001 and ns for not significative. The statistical analysis was conducted using GraphPad Prism software 10.2.2 (GraphPad Software, Boston, Massachusetts USA).

## Results

### Medical devices treatment performances

Collected blood allowed for at least two consecutive treatment cycles for each animal and each device. Cycle 1 and 2 were independently analyzed. Due to larger collected volume, a third cycle was performed on three occasions with each device (3 out of 10 for Xtra^®^ device and 3 out of 10 for same™ device). However, blood volumes were incomplete and non-representative, so data of third cycles are not shown.

A median diluted blood volume of 1090mL (882–1342) per animal was treated with the Xtra^®^ device, allowing to retrieve a median final concentrated blood volume of 245mL (238–432). A median diluted blood volume of 824mL (631–1148) per animal was treated by same™, allowing to obtain a median final concentrated blood volume of 205mL (110–417). Diluted blood volume was slightly higher for Xtra^®^ compared to the same™ (* p = 0.0108) but the concentrated blood volume was not significantly different despite a tendency (ns p = 0.06). Diluted blood from the blood collection reservoir (BCR) before treatment (TR) and concentrated blood in the transfusion bag after treatment (TB) were compared for multiple cell yields and plasma components washouts. All data are summarized in Tables 2 and 3.

**Table 2:** Autotransfusion devices performances in cell concentrations for the two successive treatment cycles.

Devices		Cycle 1		Difference between devices	Cycle 2		Difference between devices
		Xtra^®^ (n = 10)	same™ (n = 10)	p-value	Xtra^®^ (n = 10)	same™ (n = 10)	p-value
**RBC concentration (10^**6**^**/µL)****	**TR**	2.4	2.2	*ns*	2.6	**3.1**	**
		(1.8–4.2)	(1.6–3.2)		(2.0–4.1)	(2.0–4.0)	
	**TB**	9.0	7.6	*ns*	8.6	**9.3**	*
		(8.2–9.6)	(4.7–9.1)		(6.5–9.7)	(6.1–10.1)	
**Hematocrit (**%**)**	**TR**	15	16	*ns*	14	**18**	**
		(11–28)	(13–26)		(11–19)	(13–25)	
	**TB**	58	54	*ns*	49	**57**	*
		(55–61)	(42–61)		(31–58)	(46–65)	
**RBC yield **(**%**)****		82.4	76.7	*ns*	86.1	95.5	*ns*
		(44.8–83.9)	(58.4–89.4)		(78.9–106.6)	(56.4–113.2)	
**Platelet Concentration (10**^**6**^**/µL)******	**TR**	58	43	*ns*	**56**	40	**
		(34–156)	(26–71)		(38–84)	(33–71)	
	**TB**	14	**85**	****	18	**78**	***
		(8–41)	(50–113)		(12–36)	(60–139)	
**Platelet Yield **(**%**)****		5.1	**47.7**	****	7.9	**59**	***
		(3.2–12.4)	(32.7–132.6)		(5.1–13.4)	(48.4–80.0)	

The two devices were compared for cycle 1 and 2. Results are expressed as median (min-max). TR: in reservoir, before treatment cycle. TB: in transfusion bag, after treatment cycle. p-value significance is represented by stars where * is ≤0.05, ** is ≤0.01, *** ≤0.005, **** ≤0.001 and ns for not significative. Best results are highlighted in bold.

**Table 3:** Autotransfusion devices performances in cell concentrations for the two successive treatment cycles.

Devices		Cycle 1		Difference between devices	Cycle 2		Difference between devices
		Xtra^®^ (n = 10)	same™ (n = 10)	p-value	Xtra^®^ (n = 10)	same™ (n = 10)	p-value
**Heparin concentration **(IU/mL)****	**TR**	8.6	7.8	ns	**8.4**	6.6	**
		(3.5–9.5)	(6.1–9.7)		(7.6–9.3)	(4.9–8.1)	
	**TB**	0.94	**0.31**	***	0.52	0.11	ns
		(0.28–1.44)	(0–0.68)		(0–1.12)	(0–3.02)	
**Heparin washout (**%**)**		97.8	**99.4**	**	98.5	99.6	ns
		(96.8–99.2)	(96.4–100)		(97.3–100)	(85.8–100)	
**Free Hb concentration (**g/dL**)**	**TR**	0.65	**0.36**	***	0.7	**0.51**	**
		(0.37–1.81)	(0.18–0.63)		(0.37–1.03)	(0.3–0.76)	
	**TB**	0.65	1.30	ns	**0.97**	1.31	***
		(0.52–0.95)	(0.38–2.01)		(0.65–1.16)	(1.1–87)	
**Free Hb washout **(**%**)****		**92.4**	61.6	****	**84**	61.6	***
		(84.6–94.8)	(–18.1–87.5)		(51.4–91.2)	(–14.1–73.9)	
**Potassium washout **(**%**)****		**94.7**	91.5	****	**93**	91.7	*
		(92.7–96.6)	(72.9–93.6)		(90.7–94.9)	(70–93.8)	
**Calcium washout **(**%**)****		96.8	97.4	ns	96.8	97.3	ns
		(95.1–97.6)	(86.7–98.5)		(94.3–97.3)	(72.2–98.1)	
**Phosphate washout **(**%**)****		95.8	96.2	ns	96.4	96.9	ns
		(46.7–98.5)	(83.8–98.4)		(59.1–98.3)	(85.3–98.6)	
**Creatinine washout **(**%**)****		85.4	84.1	ns	83.4	86.3	ns
		(79.7–92.9)	(69.5–89.7)		(77.4–86.4)	(69.2–91.7)	
**Albumin washout **(**%**)****		94.1	92.7	ns	92.6	91.5	ns
		(81.3–96.8)	(69.5–94.7)		(80.3–94.6)	(71.4–93.8)	
**Protein washout **(**%**)****		94.6	94	ns	93.5	90.4	ns
		(63.3–97.6)	(47–96.1)		(63.6–96.1)	(63.1–96.4)	
**Glucose washout **(**%**)****		97.6	96.2	ns	96.6	96.5	ns
		(76.1–98.4)	(69.5–98.6)		(75.3–98.1)	(72.2–98.6)	
**Triglycerides washout **(**%**)****		**91.5**	85.6	**	**90.3**	81.3	*
		(67.4–93.5)	(38.4–91.7)		(62–91.9)	(58.2–90.1)	
**NEFA washout **(**%**)****		94.6	95.6	ns	90.8	92.8	ns
		(81.3–97.4)	(92.4–97.6)		(79.9–96)	(65.3–97.5)	
**Haptoglobin washout **(**%**)****		96.6	94.5	ns	96.35	88.4	ns
		(−481.1–99.8)	(−1545–99.7)		(−338.7–99.9)	(–5735–99.2)	
**LDH washout **(**%**)****		99.7	99.5	ns	99.6	99.1	ns
		(64.1–100)	(69.5–100)		(42.2–100)	(–1841–100)	
**Lactate washout **(**%**)****		88.9	87.8	ns	88.9	89.9	ns
		(86.8–93.2)	(77.2–92.4)		(85.1–94.2)	(77.8–92.6)	
**TNFalpha washout **(**%**)****		97.8	100	ns	79.85	87.3	ns
		(14.7–100)	(−11.2–100)		(−127.6–100)	(10.9–100)	
**PigMAP washout **(**%**)****		93.7	96.1	ns	93.5	92.7	ns
		(89.9–98.3)	(91.3–99.1)		(90.2–100)	(72.5–98.9)	
**CRP washout **(**%**)****		95.3	93.5	ns	89.6	94.9	ns
		(75.2–100)	(–1.7–99.4)		(76.9–99.7)	(90.4–99.9)	

The two devices are compared for cycle 1 and 2. Results are expressed as median (min-max). TR: in reservoir, before treatment cycle. TB: in transfusion bag, after treatment cycle. p-value significance is represented by stars where * is ≤0.05, ** is ≤ 0.01, *** ≤0.005, **** ≤0.001 and ns for not significative. Best results are highlighted in bold.

### RBC concentrations and yield

**Fig 2:**
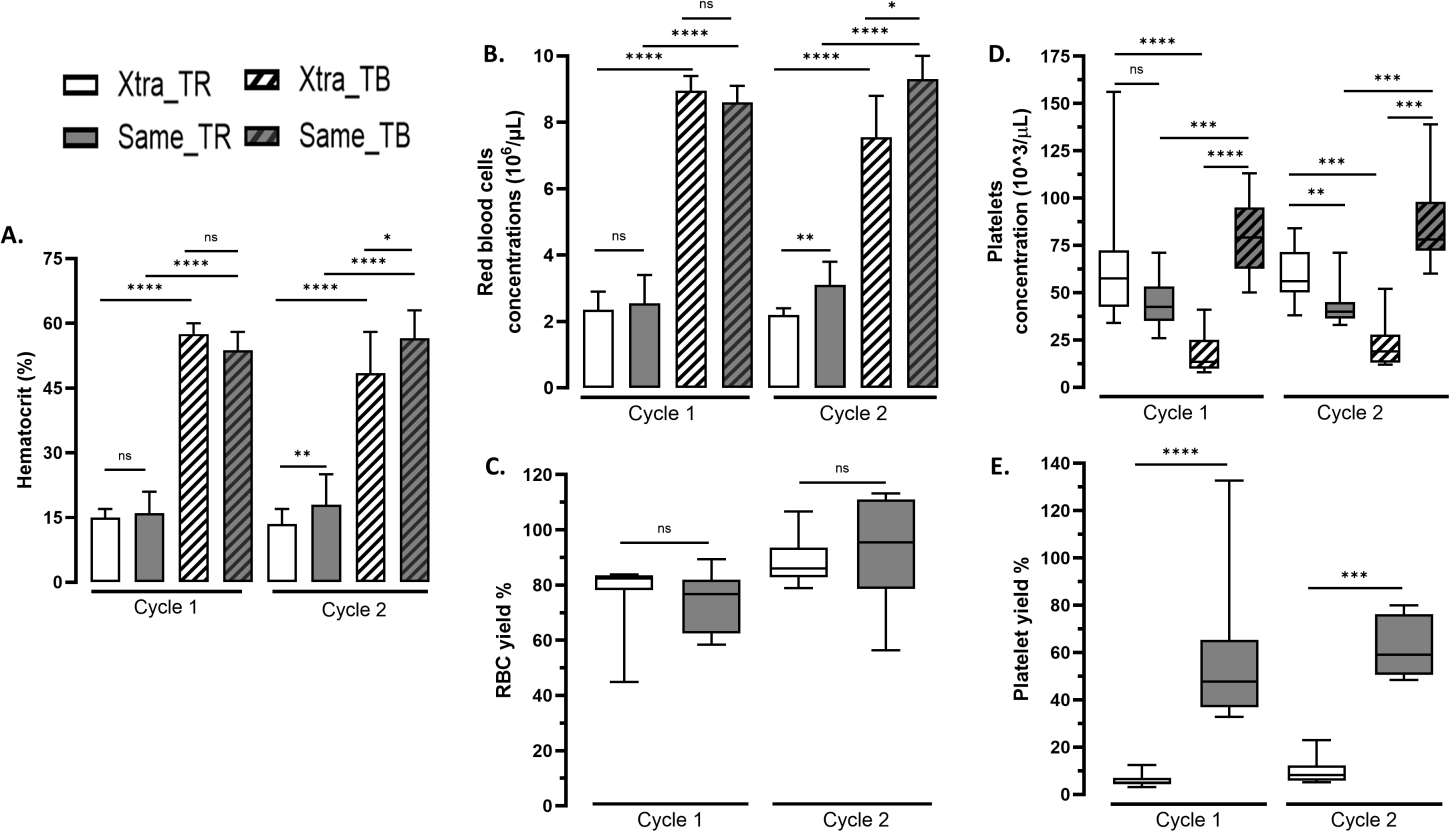
Comparative performances of the two autotransfusion devices. In white Xtra^®^ device, in gray same™ device, with stripes in transfusion bag after blood treatment, data represent median and error bars for min-max for each cycle. **A.** Hematocrit (%) in input (reservoir) and output (transfusion bag). **B.** RBC concentrations (10^6^/µL) in input (reservoir) and output (transfusion bag). **C.** Calculated RBC yield (%). **D.** Platelet concentrations (10^3^/µL) in input (reservoir) and output (transfusion bag). **E.** Calculated platelet yield (%).

Initial hematocrits and RBC concentrations of diluted shed blood were not different between groups in TR for cycle 1, and the treatment process achieved a concentrated blood in cycle 1 with similar hematocrits and RBC concentration for Xtra^®^ and same™. For cycle 2, initial hematocrit and RBC concentration in TR were lower for Xtra^®^ than same™. Cycle 2 concentrated blood in TB showed a lower hematocrit and RBC concentration for Xtra^®^ than same™. However, median hematocrits in TB were within the recommended 45–65% range [32] for both devices. Detailed results are presented in the Fig 2 and Table 2. RBC recovery performance evaluated by RBC yield were similar between devices at each cycle as presented in Fig 2.

### Platelets concentrations and yield

Initial platelet concentration in TR was not different between devices for cycle 1. Filtration-based same™ device allowed to concentrate platelets to achieve a high platelet concentration in TB, resulting in a significantly six times higher platelet concentration and nine times higher yield for same™ device than Xtra^®^ device in TB. At the cycle 2, despite a significantly lower initial concentration of platelets for same™ device compared to Xtra^®^, treatment cycle resulted in a significantly four times higher concentration and seven times higher yield of platelets by same™ device than Xtra^®^ device in TB. Detailed results are presented in the Fig 2 and Table 2.

### Washout performances

#### Heparin concentration and washout.

**Fig 3:**
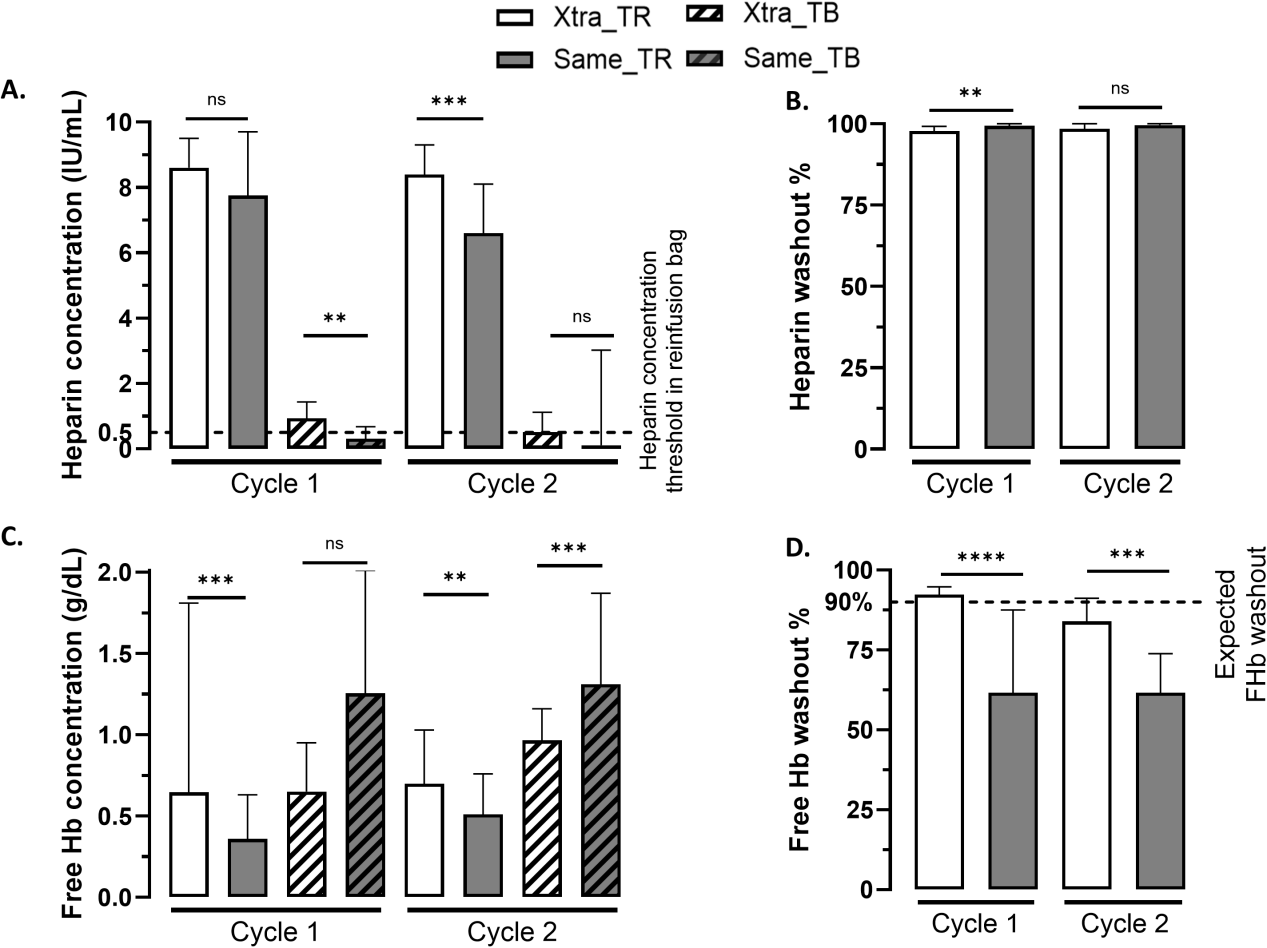
Comparative washout of the two autotransfusion devices. In white Xtra^®^ device, in gray same™ device, with stripes for transfusion bag after blood treatment, data represent median and error bar for max for each cycle. **A.** Heparin concentration (IU/mL) in input (blood collection reservoir, no stripes) and output (transfusion bag, with stripes). Threshold represented at 0.5IU/mL is corresponding to AABB requirement of maximal remaining heparin concentration in reinfusion bag. **B.** Calculated heparin washout (%). **C.** Free hemoglobin concentration (g/dL) in input (reservoir, no stripes) and output (transfusion bag, with stripes). **D.** Calculated free hemoglobin washout (%). Above a 90%-threshold of FHb washout, AABB considers a good performance.

Results are detailed in Fig 3 and Table 3. For cycle 1, heparin concentration was similar between same™ and Xtra^®^ devices in TR, decreased in TB after treatment and was significantly lower for same™ than Xtra^®^, meaning a significantly higher washout by same™ device than Xtra^®^ device. For cycle 2, heparin concentration in TR was significantly higher for Xtra^®^ than same™, decreased in TB and was not significantly different between devices, meaning a washout not significantly different between devices.

#### Free hemoglobin concentration and washout.

Plasmatic free hemoglobin concentration was significantly lower in TR for same™ device than Xtra^®^ device at cycle 1 and cycle 2. In TB, free hemoglobin concentration at cycle 1 was not different between groups but was significantly lower at cycle 2 for Xtra^®^ device than same™ with 0.97 g/dL (0.65–1.16) vs 1.31 g/dL (1.1–87) respectively, *** p = 0.0001. The calculated washout of free hemoglobin was higher with Xtra^®^ device than same™ device for cycle 1 and cycle 2 (Fig 3 and Table 3).

#### Plasmatic components washouts.

Washouts of other plasmatic components were calculated independently for cycle 1 and cycle 2 for both devices and almost all parameters were similarly washed with no statistical difference (detailed in Table 3). Briefly creatinine, haptoglobin and lactate washouts reached at least an 80% rate for both devices with no significant differences. Potassium, calcium, phosphate, albumin, total protein, glucose, NEFA, LDH, Pig-MAP and CRP washouts reached at least an 90% rate for both devices with no significant differences. Only two exceptions of significant differences were noted: potassium and triglycerides. Potassium washouts were above 90% for both devices but Xtra^®^ device showed a better performance than same™ device at cycle 1 (median Potassium washout of 94.7% vs 91.5% respectively, p < 0.0001) and at cycle 2 (median Potassium washout of 93% vs 91.7% respectively, p = 0.0141). Triglycerides washouts were above 80% for both devices but Xtra^®^ device showed a better performance than same™ device at cycle 1 (median triglycerides washout of 91.5% vs 85.6% respectively, p = 0.0064) and at cycle 2 (median triglycerides washout of 90.3% vs 81.3% respectively, p = 0.032).

### Data on animals

After device performance evaluations on processed blood, the *in vivo* part of the study compared pigs with autotransfusion, *xtra* and *same* groups, or no-transfusion *control* group during a 72-h follow-up (n = 7 for each animal group). There was no significant difference between the three groups for anesthesia duration, bleeding speed, transfusion time delay from the end of bleeding to the beginning of transfusion and total ringer lactate volume administered during anesthesia (Table 4).

**Table 4:** Anesthetic management synthesis.

ANIMAL GROUPS	*control* group (n = 7)	*xtra* group (n = 7)	*same* group (n = 7)
**Anesthesia duration (min)**	225 (155–300)	195 (170–260)	210 (160–230)
**Bleeding speed (mL/kg/h)**	11.7 (5.5–32.4)	9.7 (6.7–16.7)	7.7 (4.8–19.5)
**Blood loss volume (%)**	13.4% (8.7–26.4)	15.2% (11.6–17.4)	12.6% (9.2–25.2)
**Delay before reinfusion (min)**	No transfusion	21 (19–26)	23 (18–48)
**Intravenous crystalloid volume during anesthesia (mL/kg)**	33 (25–36)	29 (22–43)	27 (19–32)
**Number of animals with advanced IV fluid administration post-bleeding**	4 animals	0 animal	1 animal
	Colloid solution (Voluven 6%) 2.5–4 mL/kg		Hypertonic saline 10% 1mL/kg
	2 animals of the 4 Hypertonic saline 10% 1–1.5 mL/kg		
**Intravenous crystalloid volume during recovery** **(for 4 to 8 hrs)**	3 animals 13–43 mL/kg	2 animals 22–35 mL/kg	3 animals 10–18 mL/kg

Results are expressed as median (min-max).

In the no-transfusion *control* group, 57% of the animals (4/7) required advanced intravenous (IV) fluid management during anesthesia with a bolus of colloid solution and 29% (2/7) received an additional bolus of hypertonic saline with the colloids to maintain hemodynamic stability. One animal in the *same* group (14%) received a bolus of hypertonic saline. During immediate recovery, a total of 8 minipigs had slow recovery and required IV fluid administration, representing 43% in the *control* group (during 4h for 2 animals and 8h for 2 animals), 29% in *xtra* group for 4 to 5 hours and 43% in *same* group for 3 hours. Two animals, 1 *control* and 1 *xtra*, necessitated administration of nasal oxygen after extubation for a few hours. All had resumed normal behavior and appetite the following morning except one *xtra* animal that had decreased appetite, increased sleeping time and required additional fluid and energy support during the day.

#### Hematologic follow-up.

Complete hematological follow-up was performed with complete blood cell counts (CBC) during anesthesia before the surgery (T0), at the end of bleeding (TE), at the end of the transfusion (TP), then postoperatively at the four time points of the follow-up (H2, H24, H48 and H72). Table 5 presents selected parameters and detailed CBC is displayed in the supporting information, S1 Table.

**Table 5:** Selected hematological parameters in animals during the 72 h follow-up.

Parameters	Groups	T0	TE	TP	H2	H24	H48	H72
**RBC count (10** ^**6**^ **/µL)**	control	5.5	5.7	4.8	5.3	4.4	4.5	4.4
		(4.6–6)	(4.4–5.9)	(4–5.4)	(4.7–6)	(3.5–5.1)	(3.8–6.4)	(3.7–5)
	xtra	5.1	5.6	5.8**	6.2*	5.7**	5.6*	5.0**
		(4.9–6.5)	(5.2–6.1)	(5–6.3)	(5.4–6.9)	(5.2–6.1)	(4.8–5.9)	(4.9–6.1)
	same	4.8	5.5	5.5*	5.8	5.0*	5.1	5.3
		(4.4–5.6)	(4.5–6.1)	(4.2–6.3)	(5.1–6.7)	(4.3–5.8)	(4.2–5.4)	(4.4–5.9)
**Hemoglobin **(g/L)****	control	111	113	95	105	85	93	91
		(90–121)	(90–120)	(82–109)	(97–124)	(73–101)	(75–126)	(76–100)
	xtra	104	115	123**	133*	115**	110*	106*
		(103–134)	(105–124)	(102–131)	(110–147)	(105–125)	(103–122)	(97–127)
	same	102	115	117*	121*	107*	110	112
		(92–116)	(94–125)	(88–130)	(107–139)	(90–121)	(87–114)	(95–124)
**Hematocrit **(%)****	control	35	36	29	33	26	29	29
		(27–39)	(28–38)	(26–34)	(30–40)	(22–31)	(23–42)	(23–32)
	xtra	33	37	38**	42*	36***	35*	34*
		(32–44)	(34–40)	(32–41)	(35–49)	(33–39)	(32–39)	(30–40)
	same	33	37	37*	39	32**	34	34
		(28–37)	(29–40)	(27–42)	(33–45)	(28–38)	(28–37)	(30–40)
**Platelets (10^**3**^**/µL)****	control	313	269	250	275	240	261	315
		(141–434)	(217–379)	(189–326)	(207–409)	(140–385)	(151–380)	(225–446)
	xtra	337	289	254	295	309	304	332
		(104–447)	(232–362)	(182–345)	(225–417)	(256–401)	(224–428)	(139–465)
	same	422	338	333	336	319	311	344
		(239–468)	(207–390)	(218–365)	(201–388)	(217–385)	(219–361)	(265–419)
**WBC (10** ^**3**^ **/µL)**	control	9.6	8.1	9.3	13.9	19.4	15.8	15.4
		(7.7–11.3)	(8.1–11.8)	(7.5–14)	(9.4–16.5)	(17.1–22.2)	(9.3–19.2)	(9.5–18.5)
	xtra	9.6	10.6	10.7	14.7	18.7	12.9	12.6
		(6.7–19.2)	(7.8–13)	(7.9–16.2)	(9.8–15.6)	(14.1–24.7)	(11.3–17.8)	(9.8–16.3)
	same	9.7	8.3	9.2	16.3	18.6	15.4	13.9
		(8.5–12.5)	(7.3–11.3)	(6.5–12.5)	(9.6–19.8)	(13.4–24.8)	(11.1–20)	(10–19.1)
**Neutrophilic**	control	4	5.3	5.9	11.4	15.4	9.6	8.2
**granulocytes (10^3^/µL)**		(3–5.3)	(4.2–6.9)	(2.2–9.1)	(5.2–13.5)	(12.5–17.5)	(5.6–14)	(5.2–12.1)
	xtra	3.3	5.8	6.4	10.3	12.9	7	7.3
		(2.8–16.9)	(3.6–9.3)	(4.6–10.2)	(6.4–12.6)	(10.7–18.7)	(6–12.3)	(5–11.3)
	same	4.5	4.9	6	12.4	13.9	9.2	7.8
		(2.9–5.9)	(2.8–7)	(2.8–8.9)	(6.6–15.8)	(9.1–18.3)	(5.8–13.2)	(5.2–11.6)

Complete blood count available in the supporting information, S1 File.

xtra (n = 7) and same (n = 7) groups were independently compared to control group (n = 7) at the corresponding time-point. Results are expressed as median (min-max). p-value significance is represented by stars where * is ≤0.05, ** is ≤0.01, *** ≤0.005, **** ≤0.001. Significant differences from control group are highlighted in bold.

T0: baseline under anesthesia once central venous catheter in place and before the surgery start, TE: end of bleeding, TP: end of transfusion, H2: between 2 and 6 hours postoperatively, H24-H48-H72: 24-, 48- and 72-hours post-transfusion.

Right after retransfusion for *xtra* and *same* groups (TP), RBC count, hematocrit and hemoglobin were significantly higher than the no-transfusion *control* group (blood sample taken at the end of anesthesia). Parameters stayed statistically higher in the reinfused groups at all time of follow-up for *xtra* group (H2, H24, H48 and H72) and at H24 for *same* group compared to the *control* group. No difference in the WBC counts were identified between groups. All animals developed postoperatively a neutrophilic leukocytosis with a peak at H24 that started resolving between H48 and H72. No other significant difference between groups at matching times was found in the remaining CBC parameters (reticulocytes, platelets or among other white blood cells).

#### Biochemical analysis.

Analyses on animal blood samples included concentration measurement of sodium, potassium, chlore, creatinine, albumin, total proteins, glucose, triglycerides, calcium, phosphate, and NEFA. No significant difference between control and both groups of transfused animals were identified at the successive time points (supporting information, S1 Table). Follow-up of plasmatic concentrations of haptoglobin, LDH, lactate, used to assess hemolysis along with TNF-alpha, PigMAP and CRP, selected to assess inflammation, were also performed. A higher lactate concentration at the end of anesthesia was found for the *control* group (4.33 (3.43–4.81) mmol/L) compared to the end of transfusion (TP) for *same* group (2.95 (1.02–3.5) mmol/L, p = 0.0047) but only a tendency with the *xtra* group (2.8 (1.85–4.98) mmol/L, ns p = 0.0513). TNF-alpha concentration at H48 was significantly lower for *same* group and *xtra* group compared to *control* group (respectively 21.7 (15.8–47.8), p=0.0076 and 24.1 (8.8–103.3), p=0.0285 versus 46.6 (24.8–148.5) ng/L). TNF-alpha concentration was also significantly lower at H72 for *same* group compared to *control* group at H72 (respectively 19.9 (0–30.3) and 35.1 (17.3–135.7) ng/L, p = 0.0221). There was no statistically significant difference between *the 3* groups for the other parameters (haptoglobin, LDH, PigMAP and CRP).

#### Coagulation parameters.

To specifically document the effects of platelet reinfusion provided by the same™ device, coagulation marker measurements and tests were performed: D-Dimer and fibrinogen concentrations; prothrombin time (PT), activated partial thromboplastin time (APTT), ROTEM thromboelastrometry (clotting time (CT), maximal clot firmness (MCF), α-angle, clot lysis at 30 minutes (Ly30), area under the curve (AUC)). Results are presented in the Table 6. Results did not show any abnormal response and any significant differences between groups over time.

**Table 6:** Follow-up of coagulations markers (D-Dimer and fibrinogen concentrations, PT and APTT, ROTEM EXTEM).

**Parameters**	**Groups**	**T0**	**TE**	**TP**	**H2**	**H24**	**H48**	**H72**
**D-Dimer concentration** ** ** **(µg/L)**	control	0.0	22.7	12.3	0.0	0.0	0.0	0.0
		(0–146.5)	(0–48.2)	(0–111)	(0–73.9)	(0–104.8)	(0–10.9)	(0–36.1)
	xtra	0.0	0.0	7.8	17.7	17.7	0.0	7.3
		(0–39.4)	(0–326.9)	(0–336.1)	(0–356.2)	(0–331)	(0–327.9)	(0–374.3)
	same	0.0	10.4	14.8	0.0	0.0	4.9	18.0
		(0–85.7)	(0–199.6)	(0–114)	(0–149.6)	(0–57)	(0–200.8)	(0–169.9)
**Fibrinogen concentration** ** ** **(g/L)**	control	2.04	1.95	1.29	1.91	4.30	5.23	4.95
		(1.63–2.6)	(1.72–2.04)	(1.05–1.56)	(1.34–2)	(1.72–5.46)	(4.13–6.9)	(4.19–6)
	xtra	2.09	1.72	1.37	2.15	4.86	5.46	4.64
		(1.13–2.79)	(1.16–2.09)	(1–2)	(1.52–2.44)	(3.99–5.75)	(4.99–5.52)	(4.42–4.84)
	same	2.28	1.69	1.94	1.99	5.11	5.19	4.67
		(1.4–3.69)	(1.42–3.02)	(1.33–2.98)	(1.45–3.55)	(4.43–6)	(4.3–5.95)	(3.56–5.88)
**Prothrombin time (s)**	control	12	13	14	13	15	15	15
		(11–13)	(13–13)	(13–15)	(13–14)	(14–16)	(13–15)	(13–16)
	xtra	13	14	14	14	15	15	15
		(12–15)	(12–15)	(13–18)	(12–16)	(14–16)	(15–17)	(14–18)
	same	14	13	13	14	15	14	14
		(13–14)	(13–15)	(13–14)	(13–15)	(14–17)	(14–16)	(14–15)
**APTT** ** ** **(s)**	control	10.0	16.0	19.0	14.0	17.0	16.0	14.5
		(8–15)	(14–19)	(15–22)	(13–14)	(16–18)	(14.2–18)	(10–18)
	xtra	12	17	17	15	17	16	16
		(8–14)	(14–22)	(14–22)	(14–18)	(16–20)	(15–18)	(14–17)
	same	14	16	15	13	17	18	15
		(13–15)	(15–18)	(14–21)	(10–16)	(14–18)	(16–21)	(14–16)
**CT** ** ** **(s)**	control	49	55	58	59	69	64	69
		(43–72)	(51–59)	(54–78)	(51–64)	(49–82)	(61–120)	(39–80)
	xtra	48	61	60	61	66	67	72
		(36–82)	(48–65)	(49–78)	(50–73)	(53–76)	(61–77)	(62–79)
	same	52	58	52	71	65	70	64
		(43–71)	(50–64)	(49–58)	(58–78)	(61–74)	(65–75)	(53–74)
**MCF** ** ** **(mm)**	control	71	67	66	68	77	78	78
		(70–75)	(62–70)	(64–70)	(67–72)	(72–78)	(71–80)	(76–79)
	xtra	72	68	68	70	75	76	78
		(58–76)	(62–72)	(64–70)	(61–74)	(75–79)	(74–78)	(76–80)
	same	73	71	71	72	78	76	77
		(67–80)	(62–79)	(64–76)	(63–76)	(73–79)	(74–79)	(75–82)
**AUC** ** ** **(arbitrary unit)**	control	7049	6710	6568	6799	7661	7746	7787
		(6960–7375)	(6156–6980)	(6337–6932)	(6670–7133)	(7155–7781)	(7097–8011)	(7590–7875)
	xtra	7165	6754	6721	6952	7485	7616	7742
		(5823–7585)	(6194–7183)	(6360–6951)	(6098–7342)	(7465–7854)	(7446–7723)	(7539–7955)
	same	7265	7068	7028	7210	7743	7578	7677
		(6700–7891)	(6138–7869)	(6354–7531)	(6299–7597)	(7239–7850)	(7383–7925)	(7470–8134)
**α angle** ** ** **(°)**	control	77	74	75	76	76	77	78
		(74–78)	(72–77)	(71–77)	(74–77)	(73–77)	(65–78)	(74–79)
	xtra	77	75	75	76	75	76	77
		(68–80)	(72–77)	(73–76)	(65–77)	(72–78)	(71–78)	(74–80)
	same	75	77	77	75	76	76	77
		(74–80)	(70–78)	(73–78)	(70–77)	(69–78)	(73–77)	(75–78)
**Ly30** ** ** **(%)**	control	97	97	98	98	98	98	97
		(95–98)	(96–99)	(97–100)	(97–99)	(97–100)	(97–100)	(96–98)
	xtra	98	98	98	99	98	97	97
		(95–99)	(95–99)	(96–100)	(97–100)	(96–99)	(96–99)	(94–99)
	same	97	97	98	97	98	97	97
		(94–100)	(95–99)	(95–100)	(95–99)	(97–99)	(96–99)	(94–99)

Xtra (n = 7) and same (n = 7) groups were independently compared to control group (n = 7) at the corresponding time-point. Results are expressed as median (min-max). Any significant differences were found between groups.

T0: baseline under anesthesia once central venous catheter in place and before the surgery start, TE: end of bleeding, TP: end of transfusion, H2: between 2 and 6 hours postoperatively, H24-H48-H72: 24-, 48- and 72-hours post-transfusion.

#### Necropsy observations.

Macroscopic observations during thorough necropsy examination did not show any difference between the three groups regarding undesirable thrombotic events. More details of euthanasia and examination are provided in the supporting information, S1 File.

## Discussion

The aim of this study was to compare the new filtration-based same™ device and the conventional centrifugation-based Xtra^®^ device in a swine model of surgically induced controlled splenic bleeding. Pigs, and especially minipigs, have become a major translational research model over the last three decades and have replaced the dog as the general large animal surgical model in many areas of the world, with great acceptance by regulatory authorities [43,44]. A splenic bleeding model followed by an hemisplenectomy on minipigs was used to assess device performances regarding cell concentrations and undesirable component washout and animal recovery during a 72h post-operative follow-up.

Concerning autotransfusion device performance comparison, RBC concentration was equivalent between the devices during the first cycle of treatment, but RBC yield was significantly higher for same™ device compared to Xtra^®^ device during the second cycle. Regarding platelet concentration, same™ device performances were also superior to Xtra^®^ device for the final platelet numeration and yield. Indeed, platelet concentration decreased in Xtra^®^ treated blood while it significantly increased with same™. Heparin concentration in diluted blood was coherent with conventional practices and close from human heparinization in cardiac surgery [45] and heparin washout was very efficient for both devices. The blood treatment by same™ device fully respected the AABB threshold concentration of residual heparin (< 0.5 IU/mL [32]) but blood treatment by Xtra^®^ device retrieved a slightly higher median of residual heparin concentration at cycle 1, above AABB recommendation. The filtration-based autotransfusion device demonstrates cell-salvage efficacy comparable to the gold-standard centrifugation-based device, in strong agreement with AABB guidelines [31,46].

Free hemoglobin concentrations and washouts constitute the main difference between the two devices. On one hand, the initial free hemoglobin concentration in the collecting reservoir (TR), was significantly lower for *same* group suggesting less RBC lysis induced during aspiration or in the reservoir of same™ device. On the other hand, the final free hemoglobin concentration in the transfusion bag (TB), is significantly lower for *xtra* group leading to a possible explanation that Xtra^®^ treatment induces less RBC lysis or is able to better wash free hemoglobin. As all the other components were highly washed with same™ device during treatment cycles, it is advanced that the use of the hollow fiber filtration in conjunction with the known RBC fragility in swine species [40,42] may have resulted in more hemolysis. The study was undertaken with a preclinical version of the same™ device that has been since improved as demonstrated by a clinical study where people undergoing an on-pump cardiac surgery received an autotransfusion with the updated version of same™ showed a free hemoglobin concentration after treatment cycles within the AABB recommendations [31,47].

Multiple analyses were performed on animal blood samples during surgery and postoperative follow-up with only a few parameters showing statistically significant differences between *control* group and retransfused groups. One expected main difference was a higher RBC count, hemoglobin concentration and hematocrit in retransfused groups compared to *control* group right at the end of transfusion (corresponding to the end of anesthesia for control) and during the 72-h postoperative follow-up. Reinfusions with Xtra^®^ and same™ devices treated blood succeeded in compensating RBC loss during bleeding. The transient neutrophilic leukocytosis observed for the three groups, is consistent with splenic surgery [48,49], and cannot be attributed to autotransfusion, as the *control* group had the same profile. As far as inflammation and hemolysis markers are concerned, TNF-α exhibited a significantly lower concentration for the *same* group compared to *control* group. This might indicate a possible protective effect of retransfused platelets after same™ blood treatment against inflammation [50,51]. All other tested parameters were not different between conditions.

No adverse event was encountered following autotransfusion, particularly with same™ device comforting findings of the first human study [27]. As the measurement of the clotting parameters by rotational thromboelastometry did not show any abnormal response or difference between groups, it can be considered that the reinfusion of an autotransfusion blood product containing platelets did not induce any pro-coagulant effect to the animals. No *in vivo* nor post-mortem deleterious effect of retransfusion were identified with both devices and particularly considering the presence of platelets with the same™ treated blood.

The hemorrhagic shock induced in this study was not massive. Standardization of the hemorrhagic shock is difficult to obtain. The fixed volume hemorrhage model combined with a soft tissue injury [52] was chosen to comply with the variable animal body weight and the needed minimal volume to collect in the device blood collection reservoir to be able to run two treatment cycles. However, it has been shown to be more reliable than the fixed pressure model [52]. Indeed, it resulted in individual response to blood loss by variable degree and duration of hypotension, needs for fluid resuscitation and catecholamines. Lactate concentration during bleeding and transfusion showed great individual variations from being in the upper part of the normal range to highly increased. Some animals were able to maintain lactate concentration below 2 mmol/L during anesthesia and transfusion when others had increased concentration above 7 mmol/L but concentration mostly normalized in the post-operative follow-up as soon as 2 hours postoperatively confirming the mild degree of shock [52]. Adequate evaluation of autotransfusion potential side effects may be hindered in a model of severe hemorrhagic shock due to altered organic functions, decreased survival and cofounding factors brought by advanced resuscitation treatment [52]. The blood loss was significant enough to warrant, for the *control* group that was not transfused, the need for an adapted IV fluid therapy to hasten recovery and insure survival at 72h in comparison with autotransfused groups. The limited need of resuscitation did not induce any dilutional coagulopathy that would have negatively influenced the thrombogenic risk assessment of autotransfusion [52].

The controlled blood loss volume is one of the limitations of this study that may masks the beneficial effect of the platelet autotransfusion. An animal model of massive hemorrhagic shock with intense bleeding, able to induce a dilutional coagulopathy (blood loss >40–60% blood volume [52]), may highlight the autotransfused platelet benefits. Such a model imposes poor and short-term survival unless resuscitative strategies including administration of various blood products are implemented [52]. Those strategies would represent confounding factors with the benefit assessment of transfused RBC and platelets after same™ device treatment.

## Conclusions

This *in vivo* animal comparative study of controlled splenic bleeding and reinfusion allowed to describe the new autotransfusion same™ device performances in comparison with the conventional commercialized Xtra^®^ device. The same™ device showed comparable performances in terms of washout and also better performances regarding cell concentrations (RBC and platelets) without any undesirable effect during the 72-h hour post-transfusion follow-up period in pigs. Animal hematological and biochemical variables, parameters assessing inflammation or coagulation states were studied and did not show major differences from the no-transfusion *control* group and both retransfused groups after Xtra^®^ or same™ device blood treatment, except the benefit of RBC transfusion. Other studies should be designed to evaluate the transfusion clinical benefits and more specifically the transfusion of functional platelets during surgery especially in a massive hemorrhagic model.

## Supporting information

S1 File Materials and methods – additional details. (DOCX)

S1 Table Hematology and biochemistry on serial animal blood samples during the intervention and the 72h follow-up period. (DOCX)

## References

[1] Carless PA, Henry DA, Moxey AJ, O’Connell D, Brown T, Fergusson DA. Cell salvage for minimising perioperative allogeneic blood transfusion. In: Carless PA, editor. Cochrane Database of Systematic Reviews. Chichester, UK: John Wiley & Sons, Ltd; 2010.

[2] Yao Y, Li J, Wang M, Chen Z, Wang W, Lei L, et al. Improvements in blood transfusion management: cross-sectional data analysis from nine hospitals in Zhejiang, China. BMC Health Serv Res. 2018;18:856. doi: 10.1186/s12913-018-3673-x 30428874 PMC6237039

[3] Sharma DrD, Soni DrK. Effect of intra-operative cell salvage in negating the need for allogenic transfusion in patients undergoing primary hip and knee arthroplasty. National Journal of Clinical Orthopaedics. 2018;2(4):152–7. doi: 10.33545/orthor.2018.v2.i4c.88

[4] Scott B, Seifert F, Grimson R. Blood transfusion is associated with increased resource utilisation, morbidity and mortality in cardiac surgery. Ann Card Anaesth. 2008;11:15. doi: 10.4103/0971-9784.38444 18182754

[5] O’Brien SF, Yi Q-L, Fan W, Scalia V, Fearon MA, Allain J-P. Current incidence and residual risk of HIV, HBV and HCV at Canadian Blood Services. Vox Sang. 2012;103:83–6. doi: 10.1111/j.1423-0410.2012.01584.x 22289147

[6] Freedman J. The ONTraC Ontario program in blood conservation. Transfusion and Apheresis Science. 2014;50:32–3. doi: 10.1016/j.transci.2013.12.010 24388485

[7] Rohde JM, Dimcheff DE, Blumberg N, Saint S, Langa KM, Kuhn L, et al. Health care-associated infection after red blood cell transfusion. JAMA. 2014;311:1317. doi: 10.1001/jama.2014.2726 24691607 PMC4289152

[8] Walunj A, Babb A, Sharpe R. Autologous blood transfusion. Continuing Education in Anaesthesia Critical Care & Pain. 2006;6:192–6. doi: 10.1093/bjaceaccp/mkl042

[9] Tretiak R, Laupacis A, Rivière M, McKerracher K, Souêtre E. Cost of allogeneic and autologous blood transfusion in Canada. Canadian Cost of Transfusion Study Group. CMAJ. 1996;154:1501–8. 8625000 PMC1487839

[10] Amin M, Fergusson D, Wilson K, Tinmouth A, Aziz A, Coyle D, et al. The societal unit cost of allogenic red blood cells and red blood cell transfusion in Canada. Transfusion. 2004;44:1479–86. doi: 10.1111/j.1537-2995.2004.04065.x 15383022

[11] Shander A, Hofmann A, Ozawa S, Theusinger OM, Gombotz H, Spahn DR. Activity-based costs of blood transfusions in surgical patients at four hospitals. Transfusion. 2010;50(4):753–65. doi: 10.1111/j.1537-2995.2009.02518.x 20003061

[12] Meybohm P, Straub N, Füllenbach C, Judd L, Kleinerüschkamp A, Taeuber I, et al. Health economics of patient blood management: a cost-benefit analysis based on a meta-analysis. Vox Sang. 2020;115:182–8. doi: 10.1111/vox.12873 31823382

[13] Klein AA, Bailey CR, Charlton AJ, Evans E, Guckian-Fisher M, McCrossan R, et al. Association of Anaesthetists guidelines: cell salvage for peri-operative blood conservation 2018. Anaesthesia. 2018;73:1141–50. doi: 10.1111/anae.14331 29989144

[14] Boer C, Meesters MI, Milojevic M, Benedetto U, Bolliger D, von Heymann C, et al. 2017 EACTS/EACTA Guidelines on patient blood management for adult cardiac surgery. J Cardiothorac Vasc Anesth. 2018;32:88–120. doi: 10.1053/j.jvca.2017.06.026 29029990

[15] Samolyk KA, Beckmann SR, Bissinger RC. A new practical technique to reduce allogeneic blood exposure and hospital costs while preserving clotting factors after cardiopulmonary bypass: the Hemobag. Perfusion. 2005;20:343–9. doi: 10.1191/0267659105pf831oa 16363320

[16] Yarham G, Clements A, Oliver M, Morris C, Cumberland T, Bryan M, et al. Evaluating the ‘next generation’ of cell salvage - will it make a difference? Perfusion. 2011;26:263–70. doi: 10.1177/0267659111399951 21406485

[17] Paparella D, Whitlock R. Safety of Salvaged Blood and Risk of Coagulopathy in Cardiac Surgery. Semin Thromb Hemost. 2016;42:166–71. doi: 10.1055/s-0035-1569067 26838699

[18] Al-Riyami AZ, Al-Khabori M, Baskaran B, Siddiqi M, Al-Sabti H. Intra-operative cell salvage in cardiac surgery may increase platelet transfusion requirements: a cohort study. Vox Sang. 2015;109:280–6. doi: 10.1111/vox.12280 25898777

[19] Raphael J, Mazer CD, Subramani S, Schroeder A, Abdalla M, Ferreira R, et al. Society of cardiovascular anesthesiologists clinical practice improvement advisory for management of perioperative bleeding and hemostasis in cardiac surgery patients. Anesth Analg. 2019;129:1209–21. doi: 10.1213/ANE.0000000000004355 31613811

[20] van Hout FMA, Hogervorst EK, Rosseel PMJ, van der Bom JG, Bentala M, van Dorp ELA, et al. Does a platelet transfusion independently affect bleeding and adverse outcomes in cardiac surgery? Anesthesiology. 2017;126:441–9. doi: 10.1097/ALN.0000000000001518 28092320

[21] Transfusion of red blood cells, fresh frozen plasma, or platelets is associated with mortality and infection after cardiac surgery in a dose-dependent manner. Anesth Analg. 2020;130:488–97. doi: 10.1213/ANE.0000000000004528 31702696

[22] Melchior RW, Dreher M, Shade B, Chappell A, Fisher D, Rosenthal T. Comparison of three autotransfusion devices for utilization in the pediatric population. Perfusion. 2021;36:57–62. doi: 10.1177/0267659120921090 32475210

[23] Frank SM, Sikorski RA, Konig G, Tsilimigras DI, Hartmann J, Popovsky MA, et al. Clinical utility of autologous salvaged blood: a review. J Gastrointest Surg. 2020;24:464–72. doi: 10.1007/s11605-019-04374-y 31468332

[24] Seyfried TF, Gruber M, Streithoff F, Mandle RJ, Pawlik MT, Busse H, et al. The impact of bowl size, program setup, and blood hematocrit on the performance of a discontinuous autotransfusion system. Transfusion (Paris). 2017;57(3):589–98. doi: 10.1111/trf.13954 28233319

[25] Overdevest E, Lanen P, Feron J, van Hees J, Tan M. Clinical evaluation of the Sorin Xtra(R) autotransfusion system. Perfusion. 2012;27:278–83. doi: 10.1177/0267659112442222 22460925 PMC3573674

[26] Mansour A, Decouture B, Roussel M, Lefevre C, Skreko L, Picard V, et al. Combined platelet and erythrocyte salvage: evaluation of a new filtration-based autotransfusion device. Anesthesiology. 2021;135:246–57. doi: 10.1097/ALN.0000000000003820 33984126

[27] Schreiber K, Decouture B, Lafragette A, Chollet S, Bruneau M, Nicollet M, et al. A novel autotransfusion device saving erythrocytes and platelets used in a 72 h survival swine model of surgically induced controlled blood loss. PLoS One. 2022;17:e0260855. doi: 10.1371/journal.pone.0260855 35324911 PMC8947136

[28] Hooper N, Armstrong TJ. Hemorrhagic Shock. 2024. 29262047

[29] OECD. OECD series on principles of good laboratory practice and compliance monitoring. 2007. 9361450

[30] FDA. United States Food and Drug Administration Good Laboratory Practice for Non-Clinical Laboratory Studies - Code of Federal Regulations Title 21.

[31] AABB American Association of Blood Banks. Guidance for Standards for Perioperative Autologous Blood Collection and Administration. 1st ed. Bethesa; 2002.

[32] American Association of Blood Banks (AABB). Standards for Perioperative Autologous Blood Collection and Administration. 9th ed. 2021.

[33] Edqm.eu 21th edition. European Directorate for the Quality of Medicines and HealthCare of the Council of Europe (EDQM): Guide to the Preparation, Use and Quality Assurance of Blood Components. 2023.

[34] De Somer F, Bernolet H, Bouchez S, Bové T. Quality evaluation of the emergency program of three contemporary cell-washing machines. Perfusion. 2019;34:318–22. doi: 10.1177/0267659118819927 30628532

[35] Olsen AK, Hansen AK, Jespersen J, Marckmann P, Bladbjerg EM. The pig as a model in blood coagulation and fibrinolysis research. Scandinavian Journal of Laboratory Animal Science. 1999;26. doi: 10.23675/sjlas.v26i4.850

[36] Velik-Salchner C, Schnürer C, Fries D, Müssigang PR, Moser PL, Streif W, et al. Normal values for thrombelastography (ROTEM) and selected coagulation parameters in porcine blood. Thromb Res. 2006;117:597–602. doi: 10.1016/j.thromres.2005.05.015 15985284

[37] Kessler U, Grau T, Gronchi F, Berger S, Brandt S, Bracht H, et al. Comparison of porcine and human coagulation by thrombelastometry. Thromb Res. 2011;128:477–82. doi: 10.1016/j.thromres.2011.03.013 21492909

[38] Davidson AJ, Russo RM, Ferencz S-AE, Grayson JK, Williams TK, Galante JM, et al. A novel model of highly lethal uncontrolled torso hemorrhage in swine. Journal of Surgical Research. 2017;218:306–15. doi: 10.1016/j.jss.2017.06.045 28985866

[39] Gregoretti S. Suction-induced hemolysis at various vacuum pressures: implications for intraoperative blood salvage. Transfusion (Paris). 1996;36:57–60. doi: 10.1046/j.1537-2995.1996.36196190516.x 8607155

[40] McCrackin M, Swindle MM. Swine in the laboratory- chapter 1. In: Swindle MM, Smith AC, editors. Biology, Handling, Husbandry and Anatomy. CRC Press; 2015.

[41] Liumbruno GM, Bennardello F, Lattanzio A, Piccoli P, Rossetti G. Recommendations for the transfusion management of patients in the peri-operative period. II. The intra-operative period. Blood Transfus. 2011;9(2):189–217. doi: 10.2450/2011.0075-10 21527082 PMC3096863

[42] Kaplan JE, Saba TM. Platelet removal from the circulation by the liver and spleen. Am J Physiol Heart Circulatory Physiol. 1978;235:H314–20. doi: 10.1152/ajpheart.1978.235.3.H314 696842

[43] Swindle MM, Smith AC. Best practices for performing experimental surgery in swine. J Investigative Surg. 2013;26:63–71. doi: 10.3109/08941939.2012.693149 23281597

[44] Jain NC, editor. Essentials of veterinary hematology. 1st ed. Philadelphia: Wiley-Blackwell, 1993.

[45] Burman JF, Westlake AS, Davidson SJ, Rutherford LC, Rayner AS, Wright AM, et al. Study of five cell salvage machines in coronary artery surgery. Transfusion Medicine. 2002;12:173–79. doi: 10.1046/j.1365-3148.2002.00369.x 12071873

[46] Vieira SD, da Cunha Vieira Perini F, de Sousa LCB, Buffolo E, Chaccur P, Arrais M, et al. Autologous blood salvage in cardiac surgery: clinical evaluation, efficacy and levels of residual heparin. Hematol Transfus Cell Ther. 2021;43(1):1–8. doi: 10.1016/j.htct.2019.08.005 31791879 PMC7910157

[47] Mansour A, Beurton A, Godier A, Rozec B, Zlotnik D, Nedelec F, et al. Combined platelet and red blood cell recovery during on-pump cardiac surgery using same™ by i-SEP autotransfusion device: a first-in-human noncomparative study (i-TRANSEP study). Anesthesiology. 2023;139(3):287–97. doi: 10.1097/ALN.0000000000004642 37294939

[48] Toutouzas KG, Velmahos GC, Kaminski A, Chan L, Demetriades D. Leukocytosis after posttraumatic splenectomy. archives of surgery. 2002;137. doi: 10.1001/archsurg.137.8.924 12146991

[49] Wernick B, Cipriano A, Odom SR, MacBean U, Mubang RN, Wojda TR, et al. Temporal changes in hematologic markers after splenectomy, splenic embolization, and observation for trauma. Eur J Trauma Emerg Surg. 2017;43(3):399–409. doi: 10.1007/s00068-016-0679-0 27167236

[50] Nurden AT. Platelets, inflammation and tissue regeneration. Thromb Haemost. 2011;105:S13–33. doi: 10.1160/THS10-11-0720 21479340

[51] Thomas M, Storey R. The role of platelets in inflammation. Thromb Haemost. 2015;114:449–58. doi: 10.1160/TH14-12-1067 26293514

[52] Haas T, Innerhofer P, Klingler A, Wagner-Berger H, Velik-Salchner C, Streif W, et al. Re-transfusion of salvaged washed red cells improves clot formation in pigs as measured by rotational thrombelastometry (ROTEM). Eur J Anaesthesiol. 2008;25:473–78. doi: 10.1017/S0265021508003736 18298874

