# Supplementary material for: Comparative evaluation of two autotransfusion devices in a 72h survival swine model of surgically induced controlled splenic bleeding
Source: PLoS One. 2025 May 5;20(5):e0322568. doi: 10.1371/journal.pone.0322568 (PMC12052168; doi:10.1371/journal.pone.0322568)
Supplement: S1 File — (DOCX) [file pone.0322568.s001.docx]

**S1_File. Material and Methods – Additional Details**

**Statistical determination of animal number**

In determining the study's minimum sample size, a simulation approach, informed by Mansour et al. [1] was employed. Each parameter underwent analysis via a linear mixed model in a repeated measures design for every randomly simulated sample. Model adequacy was assessed through residuals normality analysis, relying on skewness and kurtosis. Varied sample sizes were experimented with to identify the minimum required to detect a significant effect in at least 96% of simulated data, adhering to a 5% significance level. Non-parametric methods were applied when normality distribution was not verified. The established minimum sample size per group was 7 animals, ensuring robust statistical analysis and result reliability.

**Equation of calculated performances**

Quality of the treated blood was assessed by measuring component concentrations (initial and residual) and calculating the washout rate with the following calculus:

$$Cpt clearance (\%) =\frac{(Initial quantity of cpt- Final quantity of cpt)}{Initial quantity of cpt} x 100$$

Where “cpt” is component, “quantity” is calculated by volume x concentration at a given point.

Cell yields performances (RBCs, Platelets) are calculated with the following formula:

$$Cell Yield (\%) = \frac{(Initial volume containing cells x initial concentration of cells)}{(Final volume containing cells x final concentration of cells)} x 100$$

The 15%-target blood loss was estimated by the following calculus:

$$Expected blood loss (mL) = \frac{(blood volume (mL) x collected blood hematocrit)}{animal hematocrit}$$

Where blood volume is obtained by multiplying the animal weight before surgery (kg) by 64,5, considering the reported blood volume in Yucatan minipig (61–68 mL of blood/kg of bodyweight [2] ) and using hematocrit comparison between animal baseline blood sample and in from the blood collecting reservoir (aspirated blood mixed with anticoagulation solution).

**Details on animals and the experimental protocol**

**Animals**

Adult female Yucatan mini-pigs (*Sus Scrofa domesticus)* were purchased from the French National Research Institute for Agriculture, Food and Environment breeding farm (INRAe Saint Gilles). Animals were housed in individual boxes and had a week of acclimation before the experiment started. They were fed with an appropriate amount of specific food, according to the breeder recommendations (Pig Food Special miniature-pigs, INRAe Saint-Gilles). Water was delivered *ad libitum*. Mini-pig environment enrichment included a chewing disc, a metallic chain, a ball and abundant bedding with dust-free wood shavings. Handlers visited individual boxes three times a day to accustom the animals to their presence and perform positive reinforcement exercises to facilitate post-operative care. To limit animal stress due to pre-anesthetic fasting, feeding times were kept constant and induction of anesthesia was done just prior to morning feeding.

**Anesthesia, analgesia and animal instrumentation**

Animals were immobilized in their living space by an intramuscular injection of ketamine (10 mg/kg), midazolam (0.5 mg/kg) and methadone (0.5 mg/kg). Once unconscious, they were transported on a stretcher to the anesthesia preparation room where an oxygen facial mask (5 L/min) was applied and intravenous access gained by inserting a 22-gauge cannula in an auricular vein. Propofol (0,5-1 mg/kg to effect) was administered intravenously to allow orotracheal intubation (8- or 8.5-mm internal diameter cuffed tube). Anesthesia was maintained with a balanced technique comprising sevoflurane (ETsevo of 1.6-2.1 %) in an oxygen/air mixture (FiO_2_ 45 %) delivered in a circle rebreathing system (Cato^®^ Anaesthesia Workstation, Draeger France) and continuous infusions of lidocaine (3 mg/kg/h), ketamine (0.5 mg/kg/h) and midazolam (0.5 mg/kg/h). Methadone (0.1-0.25 mg/kg IV) was re-dosed to effect during the procedure. Controlled ventilation was instituted to maintain normocapnia (EtCO_2_ of 35-40 mmHg) with a rate of 10-12 breaths per minute and a tidal volume of 10 mL/kg. Ventilatory parameters were adjusted according to arterial blood gas measurement results. Monitoring with a multi-parameter monitor (Carescape^TM^ B650, GE Healthcare France) included temperature, ECG, pulse oximetry, respiratory gas measurement (CO_2_, O_2_, Sevoflurane), non-invasive blood pressure during animal instrumentation and invasive blood pressure during surgery. Depth of anesthesia was assessed using classical signs of myorelaxation like absence of palpebral reflex and lack of jaw tone as well as unresponsiveness to surgical stimulation and adjusted to maintain a stable plane of anesthesia. A cut down to the right neck was performed to insert long term catheters (16G 15 cm Arrow^®^ Single lumen Central Venous Catheter) in the carotid artery and the jugular vein and left in place until the end of the experiment. The intra-arterial catheter allowed for invasive blood pressure monitoring and blood gas sampling. The jugular catheter was used to monitor central venous pressure, collect blood samples intra and post-operatively and administer the autotransfusion. Intravenous lactated ringer solution was administered during anesthesia at a rate of 2 to 5 mL/kg/h. During the induced hemorrhage, mean arterial blood pressure was maintained at 60 mmHg by adjusting depth of anesthesia, dobutamine (0.5-4 µg/kg/min) and/or norepinephrine (0.05-0.2 µg/kg/min) administration tailored to effect and increased lactated ringer rate (10-20 mL/kg/h). If blood pressure at the end of the bleeding episode or after transfusion remained unstable, hypertonic saline 10 % (1 mL/kg IV) was added to maintain normotension. If hypotension redeveloped at the end of volume replacement, a bolus of colloid (Voluven® 6% Frenesius, 1-2 up to 5 mL/kg) was administered.

Transfusion was administered by natural gravity following collected blood treatment by the autotransfusion device and was initiated once blood loss was stopped. Information on transfusion starting time and duration as well as animal hemodynamic constants were collected during anesthesia.

General anesthesia was stopped at the end of surgery or transfusion if it outlasted surgery completion. Animals were initially maintained in the operating room and monitored in sternal recumbency until return of spontaneous ventilation and brisk palpebral reflex. They were then transported to their recovery pen and kept under constant monitoring.

**Postoperative care**

Initial post-operative analgesia administered at the end of anesthesia included meloxicam (0.4 mg/kg IV), bupivacaine (2 mg/kg) infiltrated in the surgical wound at the end of surgery and buprenorphine (0.02 mg/kg IV). Meloxicam (0.4 mg/kg *per os*) was then administered once daily until euthanasia. Pain was assessed by evaluating overall animal behavior, response to human-animal interactions and careful palpation of the surgical zone every hour for the first 6 hours then four times a day until the end of the experiment. Rescue analgesia consisted of buprenorphine (0.02 mg/kg IV) and paracetamol (10 mg/kg orally).

**Postoperative follow-up**

A postoperative follow-up of 72 hours was carried out on each animal to detect any complication following the surgery and the treated blood reinfusion. A physical examination of the animal and blood sampling were performed between 2 h and 6 h postoperatively, at 24 h, 48 h and 72 h postoperatively (4 blood sampling times). Different parameters were monitored on the mini-pigs: overall behavior and attitude, presence and intensity of pain, appetite and water consumption, body temperature, heart rate, respiratory rate, non-invasive blood pressure until animals were able to stand up, surgical wound aspect and presence of any hemorrhage. Any adverse effects were recorded and any given medication was noted.

**Euthanasia and post-mortem examination**

At the end of the 72 h post-transfusion time or earlier in case of reaching a human endpoint, minipigs were anesthetized with an IV combination of ketamine (5 mg/kg), midazolam (0.5 mg/kg) and methadone (0.3 mg/kg) to be exsanguinated by transection of the abdominal aorta and the inferior vena cava. Massive hemorrhage leading to death under general anesthesia is an AVMA-approved method of euthanasia [3]. Depth of anesthesia was monitored during the process and adjusted by IV redosing of ketamine (5 mg/kg) and additional administration of propofol (0.05-0.1 mg/kg/min). Exsanguination improves macroscopic post-mortem organ evaluation, limits post-mortem clot formation in the vasculature and facilitates histologic examination especially when looking for signs of thrombosis [4]. A systematic post-mortem examination was performed on each animal that documented: - presence of any effusion into the abdominal and thoracic cavities; - gross aspect of the following organs observed in place and after their removal from the body: heart, liver, kidneys, spleen, lungs, and at least two different thoracic lymphatic nodes. Any gross change observed in these listed organs was recorded. The thrombogenic risk assessment associated with the use of the autotransfusion in this study was performed according to the ISO standard 10933-4 [5] and the application of the FDA guide: Use of International Standard ISO 10993-1, "Biological evaluation of medical devices - Part 1: Evaluation and testing within a risk management process", section thrombogenicity, published in June 2016 and updated in 2021 [6].

**References**

1. Mansour A, Decouture B, Roussel M, Lefevre C, Skreko L, Picard V, et al. Combined Platelet and Erythrocyte Salvage: Evaluation of a New Filtration-based Autotransfusion Device. Anesthesiology. 2021;135: 246–257. doi:10.1097/ALN.0000000000003820

2. McCrackin M, Swindle MM. Swine in the Laboratory- chapter 1. Biology, Handling, Husbandry and Anatomy. Swindle MM, Smith AC, editors. CRC Press; 2015. doi:10.1201/b19430

3. American Veterinary Medical Association Panel on Euthanasia. AVMA Guidelines for the euthanasia of animals: 2020 edition. 2020. Available: <https://www.avma.org/resources-tools/avma-policies/avma-guidelines-euthanasia-animals>

4. Renne RA, Everitt JI, Harkema JR, Plopper CG, Rosenbruch M. OECD Guidance Document on Histopathology for Inhalation Studies Supporting TG 412 (Subacute Inhalation Toxicity: 28-Day) and TG 413 (Subchronic Inhalation Toxicity: 90-Day). 2010. Available: <https://ntp.niehs.nih.gov/sites/default/files/iccvam/suppdocs/feddocs/oecd/oecd-gd125.pdf>

5. Standard I. ISO 10993-4: biological evaluation of medical devices part 4—selection of tests for interactions with blood. International Organization for Standardization: Geneva Switzerland, editor. 2017. Available: <https://www.iso.org/standard/63448.html>

6. Use of International Standard ISO 10993–1. Biological evaluation of medical devices-Part 1: Evaluation and testing within a risk management process” Guidance for Industry and Food and Drug Administration Staff Preface Public Comment. 2020. Available: <https://www.fda.gov/vaccines-blood-biologics/guidance-compliance-regulatory-information-biologics>
