# Supplementary material for: Comparative evaluation of two autotransfusion devices in a 72h survival swine model of surgically induced controlled splenic bleeding
Source: PLoS One. 2025 May 5;20(5):e0322568. doi: 10.1371/journal.pone.0322568 (PMC12052168; doi:10.1371/journal.pone.0322568)
Supplement: S1 Table — (DOCX) [file pone.0322568.s002.docx]

**S1 Table: Hematology and biochemistry on serial animal blood samples during the intervention and the 72h follow-up period**

**S1-a Table. Complete blood count parameters at the 7 sampling times**

(T0 : baseline under anesthesia once central venous catheter in place and before the surgery start), TE : end of bleeding, TP : end of transfusion, H2 : between 2 and 6 hours post-transfusion, H24-H48-H72 : 24, 48 and 72 hours post-transfusion)

| **Parameters** | **Groups** | **T0** | **TE** | **TP** | **H2** | **H24** | **H48** | **H72** |
| --- | --- | --- | --- | --- | --- | --- | --- | --- |
| **RBC count** | control | 5,5 | 5,7 | 4,8 | 5,3 | 4,4 | 4,5 | 4,4 |
| **(10^6^/µL)** |  | (4,6-6) | (4,4-5,9) | (4-5,4) | (4,7-6) | (3,5-5,1) | (3,8-6,4) | (3,7-5) |
|  | xtra | 5,1 | 5,6 | **5,8**** | **6,2*** | **5,7**** | **5,6*** | **5,0**** |
|  |  | (4,9-6,5) | (5,2-6,1) | (5-6,3) | (5,4-6,9) | (5,2-6,1) | (4,8-5,9) | (4,9-6,1) |
|  | same | 4,8 | 5,5 | **5,5*** | 5,8 | **5,0*** | 5,1 | 5,3 |
|  |  | (4,4-5,6) | (4,5-6,1) | (4,2-6,3) | (5,1-6,7) | (4,3-5,8) | (4,2-5,4) | (4,4-5,9) |
| **Hemoglobin** | control | 111 | 113 | 95 | 105 | 85 | 93 | 91 |
| **(g/L)** |  | (90-121) | (90-120) | (82-109) | (97-124) | (73-101) | (75-126) | (76-100) |
|  | xtra | 104 | 115 | **123**** | **133*** | **115**** | **110*** | **106*** |
|  |  | (103-134) | (105-124) | (102-131) | (110-147) | (105-125) | (103-122) | (97-127) |
|  | same | 102 | 115 | **117*** | **121*** | **107*** | 110 | 112 |
|  |  | (92-116) | (94-125) | (88-130) | (107-139) | (90-121) | (87-114) | (95-124) |
| **Hematocrit** | control | 35 | 36 | 29 | 33 | 26 | 29 | 29 |
| **(%)** |  | (27-39) | (28-38) | (26-34) | (30-40) | (22-31) | (23-42) | (23-32) |
|  | xtra | 33 | 37 | **38**** | **42*** | **36***** | **35*** | **34*** |
|  |  | (32-44) | (34-40) | (32-41) | (35-49) | (33-39) | (32-39) | (30-40) |
|  | same | 33 | 37 | **37*** | 39 | **32**** | 34 | 34 |
|  |  | (28-37) | (29-40) | (27-42) | (33-45) | (28-38) | (28-37) | (30-40) |
| **MCV** | control | 65,2 | 64,7 | 63,9 | 63,8 | 62,4 | 63,1 | 63,5 |
| **(fL)** |  | (59,1-67,4) | (63,6-65,4) | (59,1-65,8) | (60-67) | (57,9-63,5) | (58,7-66,1) | (59,6-65,5) |
|  | xtra | 64,1 | 64,7 | 64,4 | 66,9 | 64,3 | 65,3 | 64,5 |
|  |  | (62,5-70,1) | (62,1-69,8) | (61,4-69,6) | (62,8-71,6) | (61,5-67,5) | (60,9-66,8) | (61,2-67,5) |
|  | same | 66,2 | 66 | 66,4 | 66,6 | 64,8 | 65,7 | 66,0 |
|  |  | (64,5-70,6) | (65-69,8) | (64,9-70,7) | (65,5-70,4) | (61,9-68,4) | (63,2-68,7) | (62,9-68) |
| **CMH** | control | 20,4 | 20,5 | 20,5 | 20,4 | 20,4 | 20,5 | 20,4 |
| **(pg)** |  | (19,4-21) | (20-20,6) | (19,5-20,8) | (19,1-20,8) | (19,3-21,2) | (19,4-20,8) | (19,4-20,8) |
|  | xtra | 20,7 | 20,2 | 20,7 | 20,6 | 20,7 | 20,3 | 20,3 |
|  |  | (19,5-21,5) | (19,6-21,5) | (19,6-21,8) | (19,4-21,7) | (19,5-21,6) | (19,4-21,6) | (19,4-21,4) |
|  | same | 21 | 20,8 | 21,0 | 21,1 | 21 | 20,8 | 20,9 |
|  |  | (20,1-22) | (20,4-21,9) | (20,7-22,5) | (20,5-21,8) | (20,3-22,1) | (20,4-22) | (20,3-22) |
| **MCHC** | control | 31,2 | 31,4 | 32,1 | 31,8 | 33 | 32,5 | 31,9 |
| **(%)** |  | (31,1-32,8) | (31,3-31,9) | (31,5-33) | (30,9-32,1) | (32,5-33,3) | (30-33,3) | (31,2-33,2) |
|  | xtra | 31,2 | 31,3 | 31,9 | 31,2 | 32,3 | 31,9 | 31,7 |
|  |  | (30,3-32,5) | (30,6-31,8) | (31-32,5) | (29,9-32,2) | (31,3-32,5) | (30,7-32,4) | (31-32,2) |
|  | same | 31,4 | 31,1 | 31,6 | 31,1 | 32,1 | 32 | 32,1 |
|  |  | (30,7-32,9) | (30,8-32,4) | (31,2-32,4) | (31-32,4) | (31,8-33,2) | (31,6-32,5) | (30,8-32,7) |
| **Reticulocytes** | control | 32 | 52,2 | 57,5 | 51,9 | 33,8 | 71,8 | 77,4 |
| **(10^3^/µL)** |  | (24,1-84,7) | (31,7-61,5) | (27,8-91,2) | (41,2-113,4) | (16,8-73,9) | (35,6-201,6) | (45,3-127,1) |
|  | xtra | 36,1 | 83,9 | 72,2 | 55 | 43,3 | 57,3 | 54,5 |
|  |  | (22,4-59,6) | (43,4-127,8) | (33,7-112,1) | (33,8-115,7) | (11,2-93,8) | (26,7-118,3) | (29,8-114,5) |
|  | same | 62,8 | 101,6 | 84,2 | 82,1 | 62,1 | 88 | 91,3 |
|  |  | (35,7-91,3) | (39,4-151,5) | (54,8-139,8) | (45,4-116,1) | (35,1-90,6) | (50,5-111,5) | (36,2-103,7) |
| **Platelets** | control | 313 | 269 | 250 | 275 | 240 | 261 | 315 |
| **(10^3^/µL)** |  | (141-434) | (217-379) | (189-326) | (207-409) | (140-385) | (151-380) | (225-446) |
|  | xtra | 337 | 289 | 254 | 295 | 309 | 304 | 332 |
|  |  | (104-447) | (232-362) | (182-345) | (225-417) | (256-401) | (224-428) | (139-465) |
|  | same | 422 | 338 | 333 | 336 | 319 | 311 | 344 |
|  |  | (239-468) | (207-390) | (218-365) | (201-388) | (217-385) | (219-361) | (265-419) |
| **WBC** | control | 9,6 | 8,1 | 9,3 | 13,9 | 19,4 | 15,8 | 15,4 |
| **(10^3^/µL)** |  | (7,7-11,3) | (8,1-11,8) | (7,5-14) | (9,4-16,5) | (17,1-22,2) | (9,3-19,2) | (9,5-18,5) |
|  | xtra | 9,6 | 10,6 | 10,7 | 14,7 | 18,7 | 12,9 | 12,6 |
|  |  | (6,7-19,2) | (7,8-13) | (7,9-16,2) | (9,8-15,6) | (14,1-24,7) | (11,3-17,8) | (9,8-16,3) |
|  | same | 9,7 | 8,3 | 9,2 | 16,3 | 18,6 | 15,4 | 13,9 |
|  |  | (8,5-12,5) | (7,3-11,3) | (6,5-12,5) | (9,6-19,8) | (13,4-24,8) | (11,1-20) | (10-19,1) |
| **Neutrophilic** | control | 4 | 5,3 | 5,9 | 11,4 | 15,4 | 9,6 | 8,2 |
| **granulocytes** |  | (3-5,3) | (4,2-6,9) | (2,2-9,1) | (5,2-13,5) | (12,5-17,5) | (5,6-14) | (5,2-12,1) |
| **(10^3^/µL)** | xtra | 3,3 | 5,8 | 6,4 | 10,3 | 12,9 | 7 | 7,3 |
|  |  | (2,8-16,9) | (3,6-9,3) | (4,6-10,2) | (6,4-12,6) | (10,7-18,7) | (6-12,3) | (5-11,3) |
|  | same | 4,5 | 4,9 | 6 | 12,4 | 13,9 | 9,2 | 7,8 |
|  |  | (2,9-5,9) | (2,8-7) | (2,8-8,9) | (6,6-15,8) | (9,1-18,3) | (5,8-13,2) | (5,2-11,6) |
| **Lymphocytes** | control | 4,4 | 3,4 | 3,2 | 3,4 | 3,3 | 3,8 | 4,5 |
| **(10^3^/µL)** |  | (3,5-6) | (2,5-4,1) | (2,6-4,7) | (1,7-3,9) | (2,2-4,4) | (3,2-4,5) | (3,2-5,7) |
|  | xtra | 5,1 | 3,8 | 3,5 | 3 | 3,8 | 3,7 | 3,8 |
|  |  | (1,3-6,9) | (3,1-5,8) | (2,5-5,3) | (2,1-3,8) | (2,4-4,5) | (3-5,4) | (2,6-4,9) |
|  | same | 4,4 | 3,6 | 3,3 | 2,8 | 3,5 | 3,9 | 4,3 |
|  |  | (3,8-5,4) | (2,7-4,6) | (3-3,7) | (1,9-3,5) | (2,8-4,7) | (3-5,4) | (3,2-5) |
| **Monocytes** | control | 0,6 | 0,4 | 0,4 | 0,4 | 1,2 | 1,6 | 1,6 |
| **(10^3^/µL)** |  | (0,6-1) | (0,2-0,6) | (0,3-0,5) | (0,2-1) | (0,4-1,4) | (0,3-2,1) | (0,8-2,3) |
|  | xtra | 0,8 | 0,5 | 0,4 | 0,5 | 1 | 1,2 | 1,2 |
|  |  | (0,4-1) | (0,3-0,7) | (0,2-0,7) | (0,3-0,6) | (0,7-1,3) | (1,2-1,8) | (0,6-1,2) |
|  | same | 0,9 | 0,4 | 0,4 | 0,5 | 1,3 | 1,6 | 1,4 |
|  |  | (0,6-1) | (0,3-0,7) | (0,2-0,5) | (0,2-1,2) | (1,1-1,6) | (0,5-2,4) | (1,1-2,4) |
| **Eosinophilic** | control | 0,3 | 0,1 | 0,1 | 0 | 0,1 | 0,4 | 0,3 |
| **granulocytes** |  | (0,1-0,4) | (0,1-0,2) | (0-0,4) | (0-0,1) | (0-0,4) | (0-0,6) | (0,2-0,9) |
| **(10^3^/µL)** | xtra | 0,2 | 0,1 | 0 | 0 | 0,2 | 0,7 | 0,3 |
|  |  | (0-0,4) | (0-0,2) | (0-0,1) | (0-0,1) | (0,1-0,7) | (0,1-1) | (0-0,7) |
|  | same | 0,3 | 0,1 | 0 | 0 | 0,2 | 0,5 | 0,5 |
|  |  | (0,1-0,4) | (0-0,3) | (0-0,1) | (0-0,2) | (0,2-0,5) | (0,4-0,8) | (0,3-0,9) |
| **Basophilic** | control | 0 | 0 | 0 | 0 | 0 | 0 | 0 |
| **granulocytes** |  | (0-0) | (0-0) | (0-0) | (0-0) | (0-0) | (0-0) | (0-0) |
| **(10^3^/µL)** | xtra | 0 | 0 | 0 | 0 | 0 | 0 | 0 |
|  |  | (0-0) | (0-0) | (0-0) | (0-0) | (0-0) | (0-0) | (0-0) |
|  | same | 0 | 0 | 0 | 0 | 0 | 0 | 0 |
|  |  | (0-0) | (0-0) | (0-0) | (0-0) | (0-0) | (0-0) | (0-0) |

*Results are expressed as median (min-max). xtra (n=7) and same (n=7) groups were independently compared to control group (n=7) at the corresponding time-point.* *Results are expressed as median (min-max). p-value significance is represented by stars where * is ≤0.05, ** is ≤0.01, *** ≤0.005, **** ≤0.001. Significant differences are highlighted in bold.*

**S1-b Table. Animal biochemical profiles at the 7 sampling times**

(T0 : baseline under anesthesia once central venous catheter in place and before the surgery start), TE : end of bleeding, TP : end of transfusion, H2 : between 2 and 6 hours post-transfusion, H24-H48-H72 : 24, 48 and 72 hours post-transfusion)

| **Parameters** | **Groups** | **T0** | **TE** | **TP** | **H2** | **H24** | **H48** | **H72** |
| --- | --- | --- | --- | --- | --- | --- | --- | --- |
| **Sodium concentration** | control | 136,1 | 137,5 | 137,75 | 135,7 | 136,8 | 138,9 | 140,1 |
|  |  | (129,7 - 143,7) | (133,8 - 143,6) | (118,3 - 141,4) | (117 - 143,3) | (113 - 142,7) | (127,9 - 147) | (137 - 146,6) |
|  | xtra | 138,1 | 136,2 | 136,2 | 137 | 136,8 | 138,2 | 141,5 |
| **(mmol/L)** |  | (133,1 - 142,2) | (128,1 - 139,5) | (128,9 - 142,3) | (129,5 - 144,4) | (129,4 - 144) | (134,7 - 143,3) | (132,6 - 148,9) |
|  | same | 134,4 | 135,3 | 136,6 | 136,5 | 136,1 | 139,8 | 140,8 |
|  |  | (126,3 - 140,4) | (134,2 - 137,6) | (129,5 - 141,8) | (129,9 - 142,7) | (112,4 - 142,2) | (132,4 - 144) | (128,6 - 150,2) |
| **Potassium concentration** | control | 3,75 | 3,59 | 3,69 | 3,88 | 3,47 | 3,89 | 3,96 |
|  |  | (3,45 - 3,9) | (3,59 - 3,75) | (2,86 - 4,12) | (3,46 - 4,25) | (2,64 - 3,81) | (3,37 - 6,68) | (3,84 - 4,2) |
|  | xtra | 3,69 | 3,48 | 3,29 | 4,02 | 3,47 | 3,8 | 3,93 |
| **(mmol/L)** |  | (3,22 - 5,53) | (3,11 - 4,5) | (3,19 - 4,61) | (3,82 - 4,73) | (3,01 - 3,94) | (3,14 - 4,13) | (3,59 - 4,33) |
|  | same | 3,78 | 3,68 | 4,23 | 3,98 | 3,48 | 3,81 | 4,06 |
|  |  | (3,14 - 3,91) | (3,3 - 4,23) | (3,71 - 4,77) | (3,62 - 4,53) | (2,66 - 4,24) | (3,44 - 4,18) | (3,82 - 4,64) |
| **Chlore concentration** | control | 102,5 | 107,1 | 104,4 | 100,6 | 101,6 | 103,2 | 106,7 |
|  |  | (99,4 - 105,1) | (103,5 - 108,7) | (92,2 - 108,3) | (88,2 - 107,1) | (85,1 - 103,8) | (100,2 - 107,5) | (100,9 - 109,6) |
|  | xtra | 101,3 | 103,0 | 104,5 | 101,2 | 101,3 | 104,5 | 104,3 |
| **(mmol/L)** |  | (100 - 105,7) | (95,8 - 107,5) | (101,5 - 108,7) | (99,3 - 105,3) | (96,3 - 104,4) | (100,1 - 106,9) | (101,4 - 108,3) |
|  | same | 100,4 | 103,3 | 103,4 | 101,6 | 100,7 | 105,1 | 103,7 |
|  |  | (96,4 - 106,7) | (100,6 - 106,3) | (98,5 - 107,2) | (97,7 - 105,9) | (84,6 - 103,4) | (101,2 - 108) | (99 - 110,9) |
| **creatinine concentration** | control | 11,60 | 11,47 | 9,63 | 11,05 | 10,42 | 10,17 | 9,19 |
|  |  | (7,01 - 13,37) | (7,37 - 11,68) | (6,11 - 11,98) | (6,64 - 13,52) | (7,24 - 14,22) | (6,29 - 12,19) | (6,75 - 12,12) |
|  | xtra | 11,19 | 9,25 | 9,36 | 10,66 | 10,34 | 9,63 | 10,17 |
| **(mg/L)** |  | (8,18 - 18,84) | (5,86 - 13,13) | (6,54 - 13,85) | (7,7 - 15,64) | (7,84 - 13,5) | (7,47 - 11,27) | (7,16 - 12,47) |
|  | same | 9,02 | 8,67 | 8,46 | 10,73 | 10,02 | 8,97 | 9,61 |
|  |  | (8,06 - 14,97) | (6,46 - 12,71) | (7,14 - 15,55) | (7,75 - 17,23) | (4,71 - 14,21) | (7,59 - 13,2) | (6,86 - 13,32) |
| **albumin concentration** | control | 28,99 | 23,79 | 20,475 | 26,5 | 25,89 | 30,35 | 33,73 |
|  |  | (19,57 - 40,41) | (22,84 - 28,98) | (16,56 - 25,19) | (12,28 - 34,35) | (23,23 - 40,93) | (24,5 - 40,28) | (29,81 - 39,5) |
|  | xtra | 29,93 | 19,55 | 22,85 | 29,45 | 29,68 | 31,34 | 31,91 |
| **(g/L)** |  | (24,24 - 40,07) | (15,59 - 31,48) | (16,92 - 30,5) | (22,4 - 37,77) | (21,02 - 35,61) | (23,61 - 35,93) | (27,4 - 38,44) |
|  | same | 28,26 | 25,63 | 24,63 | 29,44 | 27 | 31,84 | 30,65 |
|  |  | (18,89 - 38,45) | (6,36 - 31,33) | (13,87 - 32,86) | (27,89 - 35,15) | (22,04 - 37,92) | (25,5 - 39,87) | (25,47 - 42,12) |
| **total proteins concentration** | control | 53,3 | 46,1 | 35,9 | 43,9 | 44,1 | 54,1 | 63,2 |
|  |  | (34,2 - 77,1) | (40,4 - 59,6) | (29 - 44,3) | (21,7 - 60,7) | (41,7 - 70,5) | (42,9 - 74,3) | (55,1 - 73,7) |
|  | xtra | 52,1 | 35,7 | 43,6 | 54,6 | 48,8 | 57,3 | 66,9 |
| **(g/L)** |  | (43,4 - 70,9) | (30,2 - 60,1) | (31,8 - 57) | (39,2 - 70,1) | (38,8 - 68,1) | (40,2 - 70,9) | (50,7 - 72,7) |
|  | same | 53,2 | 49,8 | 47,3 | 52,4 | 44,9 | 59,7 | 58,1 |
|  |  | (34,3 - 75,6) | (11,4 - 61,1) | (25,8 - 64,5) | (46,3 - 66,7) | (41,4 - 74,1) | (46,7 - 79,2) | (46,9 - 82,5) |
| **Glucose concentration** | control | 0,64 | 1,44 | 0,91 | 1,07 | 0,69 | 0,89 | 0,84 |
|  |  | (0,32 - 1,3) | (0,7 - 1,78) | (0,63 - 1,29) | (0,42 - 1,63) | (0,48 - 0,92) | (0,51 - 3,03) | (0,64 - 0,99) |
|  | xtra | 0,55 | 0,84 | 0,73 | 0,83 | 0,69 | 0,76 | 0,87 |
| **(g/L)** |  | (0,49 - 2,29) | (0,49 - 1,66) | (0,5 - 1,3) | (0,73 - 1,32) | (0,53 - 0,9) | (0,58 - 0,93) | (0,68 - 0,95) |
|  | same | 0,61 | 0,76 | 1,00 | 0,85 | 0,57 | 0,75 | 0,77 |
|  |  | (0,33 - 0,8) | (0,51 - 0,95) | (0,38 - 1,15) | (0,77 - 1,12) | (0,48 - 0,88) | (0,61 - 0,88) | (0,46 - 1,09) |
| **Triglycerides concentration** | control | 0,51 | 0,19 | 0,23 | 0,35 | 0,21 | 0,36 | 0,57 |
|  |  | (0,23 - 0,57) | (0,17 - 0,24) | (0,12 - 0,48) | (0,2 - 0,51) | (0,12 - 0,34) | (0,25 - 0,65) | (0,48 - 0,97) |
|  | xtra | 0,56 | 0,23 | 0,40 | 0,38 | 0,17 | 0,36 | 0,47 |
| **(g/L)** |  | (0,25 - 1,1) | (0,15 - 0,37) | (0,12 - 0,6) | (0,19 - 0,58) | (0,12 - 0,39) | (0,24 - 0,54) | (0,28 - 0,97) |
|  | same | 0,32 | 0,23 | 0,43 | 0,29 | 0,18 | 0,37 | 0,47 |
|  |  | (0,23 - 0,74) | (0,14 - 0,48) | (0,23 - 0,66) | (0,2 - 0,34) | (0,13 - 0,39) | (0,31 - 0,55) | (0,39 - 0,68) |
| **Calcium concentration** | control | 81,48 | 81,92 | 80,57 | 81,57 | 72,27 | 82,36 | 93,38 |
|  |  | (54,17 - 116,63) | (60,82 - 91,72) | (41,86 - 102,19) | (43,48 - 103,73) | (62,16 - 99,55) | (65,49 - 106,81) | (82,81 - 107,79) |
|  | xtra | 80,91 | 65,43 | 72,05 | 84,69 | 77,49 | 86,74 | 96,27 |
| **(mg/L)** |  | (69,86 - 114,44) | (57,44 - 98,37) | (57,89 - 99,54) | (65,39 - 103,48) | (57,65 - 96,43) | (60,92 - 94,4) | (78,19 - 106,39) |
|  | same | 75,72 | 76,32 | 77,66 | 81,13 | 66,35 | 84,09 | 86,51 |
|  |  | (58,46 - 101,95) | (35,3 - 99,46) | (49,71 - 98,51) | (72,67 - 97,67) | (54,36 - 92,37) | (70,7 - 97,77) | (55,51 - 102,49) |
| **Phosphate concentration** | control | 65,85 | 55,12 | 52,07 | 51,93 | 53,46 | 64,04 | 64,62 |
|  |  | (44,95 - 73,22) | (52,67 - 56,82) | (38,53 - 67,39) | (35,82 - 80,34) | (42,04 - 63,62) | (52,84 - 76,41) | (53,29 - 77,84) |
|  | xtra | 68,85 | 51,65 | 53,52 | 64,89 | 53,26 | 62,61 | 64,60 |
| **(mg/L)** |  | (56,26 - 104,99) | (40,68 - 60,41) | (45,16 - 63,5) | (55,71 - 69,37) | (44,96 - 78,94) | (54,08 - 65,2) | (56,75 - 73,49) |
|  | same | 58,23 | 54,33 | 59,70 | 64,77 | 48,94 | 60,98 | 62,37 |
|  |  | (43,76 - 78,77) | (34,23 - 73,76) | (40,48 - 75,64) | (58,16 - 75,15) | (39,03 - 67,34) | (55,53 - 71,48) | (51,99 - 78,83) |
| **NEFA concentration** | control | 0,19 | 0,48 | 0,46 | 0,28 | 0,57 | 0,46 | 0,40 |
|  |  | (0,1 - 0,34) | (0,4 - 0,63) | (0,21 - 0,67) | (0,17 - 0,46) | (0,19 - 0,91) | (0,29 - 0,66) | (0,19 - 0,76) |
|  | xtra | 0,20 | 0,36 | 0,56 | 0,39 | 0,54 | 0,44 | 0,43 |
| **(mmol/L)** |  | (0,1 - 0,67) | (0,24 - 0,55) | (0,23 - 0,94) | (0,2 - 0,68) | (0,2 - 1,15) | (0,2 - 0,56) | (0,36 - 0,58) |
|  | same | 0,22 | 0,44 | 0,61 | 0,23 | 0,70 | 0,63 | 0,58 |
|  |  | (0,13 - 0,67) | (0,21 - 0,65) | (0,35 - 1,05) | (0,18 - 0,61) | (0,39 - 0,85) | (0,36 - 0,84) | (0,34 - 0,94) |
| **Haptoglobin concentration** | control | 0,7 | 1,2 | 0,4 | 0,4 | 1,4 | 1,5 | 1,7 |
|  |  | (0,29 - 1,15) | (0,04 - 1,3) | (0,1 - 0,73) | (0,08 - 0,96) | (0,74 - 1,9) | (1,16 - 1,95) | (1,16 - 2,3) |
|  | xtra | 0,9 | 0,3 | 0,5 | 0,6 | 1,6 | 1,7 | 1,6 |
| **(g/L)** |  | (0,43 - 1,77) | (0,2 - 1,33) | (0,16 - 1,6) | (0,19 - 1,69) | (0,88 - 1,79) | (1,26 - 2,15) | (1,43 - 2,5) |
|  | same | 1,1 | 1 | 0,8 | 0,9 | 1,6 | 1,7 | 1,7 |
|  |  | (0,58 - 1,65) | (0,01 - 1,76) | (0,19 - 1,75) | (0,42 - 1,71) | (1,13 - 1,8) | (1,24 - 1,85) | (1,25 - 2,09) |
| **LDH concentration** | control | 403 | 499 | 459 | 1087 | 875 | 784 | 947,5 |
|  |  | (289 - 739) | (423 - 597) | (293 - 691) | (372 - 1200) | (552 - 1200) | (605 - 1200) | (716 - 1200) |
|  | xtra | 640 | 692 | 495 | 1200 | 1010 | 1069 | 886 |
| **(U/L)** |  | (305 - 833) | (260 - 1200) | (338 - 935) | (668 - 1200) | (534 - 1200) | (443 - 1200) | (567 - 1200) |
|  | same | 698 | 751 | 983 | 1200 | 952 | 918 | 900 |
|  |  | (290 - 986) | (82 - 1200) | (267 - 1200) | (596 - 1200) | (462 - 1200) | (520 - 1200) | (446 - 1200) |
| **Lactate concentration** | control | 2,02 | 3,78 | 4,33 | 1,81 | 1,09 | 1,15 | 1,42 |
|  |  | (1,77 - 4,22) | (3,31 - 4,81) | (3,43 - 4,81) | (1,15 - 3,42) | (0,77 - 2,28) | (0,87 - 11) | (0,95 - 5,12) |
|  | xtra | 2,38 | 3,97 | 2,98 | 1,61 | 1,23 | 1,09 | 1,34 |
| **(mmol/L)** |  | (1,59 - 3,4) | (2,48 - 6,95) | (1,85 - 4,87) | (0,9 - 7,83) | (0,94 - 2,73) | (0,7 - 2,48) | (1,01 - 3,38) |
|  | same | 2,03 | 3,44 | **2,95***** | 2,3 | 1,24 | 1,06 | 1,4 |
|  |  | (1,52 - 4,87) | (2,2 - 4,11) | (1,02 - 3,5) | (0,94 - 3,8) | (0,83 - 2,88) | (0,76 - 2,28) | (0,62 - 5,05) |
| **TNF-alpha concentration** | control | 48,7 | 38,5 | 34,1 | 51,8 | 30,3 | 46,6 | 35,1 |
|  |  | (27,2 - 145,2) | (32 - 133,5) | (0 - 255,8) | (33,3 - 129,7) | (15,4 - 132,2) | (24,8 - 148,5) | (17,3 - 135,7) |
|  | xtra | 23,7 | 30,6 | 26,8 | 40,8 | 44,3 | **24,1*** | 40 |
| **(ng/L)** |  | (0 - 116,4) | (12,1 - 99,4) | (11,4 - 231,6) | (23,7 - 117,9) | (12,4 - 123,3) | (8,8 - 103,3) | (2,3 - 173,5) |
|  | same | 23,7 | 21,6 | 33,7 | 54,4 | 33,4 | **21,7**** | **19,9*** |
|  |  | (14,5 - 59,8) | (12 - 52,3) | (17,3 - 59,8) | (35,7 - 128,6) | (0 - 54,4) | (15,8 - 47,8) | (0 - 30,3) |
| **PigMAP concentration** | control | 49,5 | 46,7 | 44,3 | 50,3 | 61,9 | 59 | 50,3 |
|  |  | (34,1 - 55,1) | (34,3 - 48,6) | (36 - 58,5) | (42,2 - 80,6) | (39,6 - 67,6) | (42,5 - 73,6) | (47 - 102,9) |
|  | xtra | 45,4 | 47,1 | 40 | 52,4 | 50,3 | 50,2 | 48,2 |
| **(µg/L)** |  | (39,5 - 68,6) | (30,2 - 63,1) | (28,9 - 60,9) | (32,9 - 72) | (35,8 - 69,3) | (37,9 - 68,8) | (33,1 - 71,1) |
|  | same | 46,8 | 49,7 | 56,2 | 68,4 | 60,1 | 58,9 | 58,7 |
|  |  | (27,6 - 100,6) | (27 - 81,7) | (42,5 - 81,3) | (47,2 - 80,6) | (55,4 - 99) | (55,9 - 106,7) | (53,3 - 90) |
| **CRP concentration** | control | 151 | 245,8 | 176,2 | 288,7 | 517,3 | 450,1 | 690,3 |
|  |  | (65,9 - 277,9) | (108,7 - 305,9) | (135,6 - 330,5) | (96,1 - 495,7) | (158,7 - 705,4) | (372,5 - 701,5) | (405,2 - 806,6) |
|  | xtra | 133 | 146,9 | 154,1 | 179,7 | 413,8 | 593,1 | 501,6 |
| **(mg/L)** |  | (115,6 - 520,3) | (102 - 877,3) | (94,9 - 519,8) | (127,2 - 436,5) | (295,4 - 734,8) | (385,5 - 910) | (347,8 - 1360,9) |
|  | same | 170,3 | 189 | 210,8 | 175,6 | 354,2 | 562,3 | 640,5 |
|  |  | (108,9 - 360,1) | (56,8 - 265,5) | (107,9 - 280,9) | (111,5 - 264,5) | (292,1 - 623,5) | (348,2 - 698,3) | (410,2 - 969,4) |

*Results are expressed as median (min-max). xtra (n=7) and same(n=7) groups were independently compared to control group (n=7) at the corresponding time-point.* *Results are expressed as median (min-max). p-value significance is represented by stars where * is ≤0.05, ** is ≤0.01, *** ≤0.005, **** ≤0.001. Significant differences are highlighted in bold.*
