# Supplementary material for: Comparative evaluation of two autotransfusion devices in a 72h survival swine model of surgically induced controlled splenic bleeding
Source: PLoS One. 2025 May 5;20(5):e0322568. doi: 10.1371/journal.pone.0322568 (PMC12052168; doi:10.1371/journal.pone.0322568)
Supplement: Attachment — Submitted filename: Response To Reviewers.docx [file pone.0322568.s004.docx]

**Revision Letter**

PONE-D-24-33101

Comparative evaluation of two autotransfusion devices in a 72h survival swine model of surgically induced controlled splenic bleeding

PLOS ONE

**Response to the academic editor**

Stephen Emilio Njolomole, MB,BS ,MPH
Guest Editor
PLOS ONE

Journal Requirements:

When submitting your revision, we need you to address these additional requirements.

1.Please ensure that your manuscript meets PLOS ONE's style requirements, including those for file naming.

We have reviewed the style requirements and have implemented required changes.

2. Thank you for stating the following in the Competing Interests/Financial Disclosure* (delete as necessary) section:

a. Please provide an amended Funding Statement declaring this commercial affiliation, as well as a statement regarding the Role of Funders in your study. If the funding organization did not play a role in the study design, data collection and analysis, decision to publish, or preparation of the manuscript and only provided financial support in the form of authors' salaries and/or research materials, please review your statements relating to the author contributions, and ensure you have specifically and accurately indicated the role(s) that these authors had in your study. You can update author roles in the Author Contributions section of the online submission form.

Please find below the updated and completed funding statement

The study was funded by BPi France (French Public Bank for investments, https://www.bpifrance.com/) by the grant PSPC-402492 (BPI grants for structuring competitive research and development). The grant was awarded to the consortium Isep/Oniris/Université de Rennes (all the authors).

The funders had no role in study design, data collection and analysis, decision to

publish, or preparation of the manuscript. The funder provided support in the form of salaries for authors [BD, CL, MB, MD, PF-V] and participated in covering the cost of animal experimentations, but did not have any additional role in the study design, data collection and analysis, decision to publish, or preparation of the manuscript. The specific roles of these authors are articulated in the ‘author contributions’ section

b. Please also provide an updated Competing Interests Statement declaring this commercial affiliation along with any other relevant declarations relating to employment, consultancy, patents, products in development, or marketed products, etc.

Within your Competing Interests Statement, please confirm that this commercial affiliation does not alter your adherence to all PLOS ONE policies on sharing data and materials by including the following statement: "This does not alter our adherence to  PLOS ONE policies on sharing data and materials.” (as detailed online in our guide for authors http://journals.plos.org/plosone/s/competing-interests) . If this adherence statement is not accurate and there are restrictions on sharing of data and/or materials, please state these. Please note that we cannot proceed with consideration of your article until this information has been declared.

Updated competing interests statement

Chloé Libaud (CL) is employed as a research and development engineer by i-SEP (Nantes, France).

Dr. Benoit Decouture (BD) was employed as project manager by i-SEP (Nantes, France).

Dr. Mallorie Depond (MD) is currently employed as project manager by i-SEP (Nantes, France), she joined the company after the data acquisition and completion of the study.

Marine Bruneau (MB) was employed as a research and development engineer by i-SEP (Nantes, France).

Dr. Patricia Forest-Villegas (PVF) is currently employed as scientific director by i-SEP (Nantes, France).

The authors confirm the fact that some of the co-authors are employed by i-SEP does

not alter their adherence to PLOS ONE policies on sharing data and materials. These affiliations do not influence the objectivity or integrity of the research presented in this manuscript All data generated during the study are fully available and all relevant data have been incorporated into the manuscript.

The evaluated technology is patented by the manufacturer i-SEP. The commercial version of the system evaluated in this study has been available on the European market since July 2022.

Please include both an updated Funding Statement and Competing Interests Statement in your cover letter. We will change the online submission form on your behalf.

The statements have been incorporated in our cover letter.

3. We note that your Data Availability Statement is currently as follows: “All relevant data are within the manuscript and in Supporting Information files.”

Please confirm at this time whether or not your submission contains all raw data required to replicate the results of your study. Authors must share the “minimal data set” for their submission. PLOS defines the minimal data set to consist of the data required to replicate all study findings reported in the article, as well as related metadata and methods (https://journals.plos.org/plosone/s/data-availability#loc-minimal-data-set-definition).

If your submission does not contain these data, please either upload them as Supporting Information files or deposit them to a stable, public repository and provide us with the relevant URLs, DOIs, or accession numbers. For a list of recommended repositories, please see https://journals.plos.org/plosone/s/recommended-repositories.

Raw data files have been added to the submission of the revised manuscript

4. Please include your full ethics statement in the ‘Methods’ section of your manuscript file. In your statement, please include the full name of the IRB or ethics committee who approved or waived your study, as well as whether or not you obtained informed written or verbal consent. If consent was waived for your study, please include this information in your statement as well.

Full ethics statement has been moved from the supplemental file to the beginning of the Methods section.

5. Please include captions for your Supporting Information files at the end of your manuscript, and update any in-text citations to match accordingly. Please see our Supporting Information guidelines for more information: <http://journals.plos.org/plosone/s/supporting-information>.

The list of supplemental information files and names has been added at the end of the manuscript just before the references

6. Please review your reference list to ensure that it is complete and correct. If you have cited papers that have been retracted, please include the rationale for doing so in the manuscript text, or remove these references and replace them with relevant current references. Any changes to the reference list should be mentioned in the rebuttal letter that accompanies your revised manuscript. If you need to cite a retracted article, indicate the article’s retracted status in the References list and also include a citation and full reference for the retraction notice.

The reference list has been checked.

Additional Editor Comments:

***Compliment***

The paper „Comparative evaluation of two autotransfusion devices in a 72h survival swine model of surgically induced controlled visceral blood loss“ represents an original work in its content. The paper is easy to read, with clearly defined objectives, detailed methodology. The results are clearly presented. I enjoyed reading the paper.
Good luck with the publication

Thank you very much for your comments

***Compliment and Questions***

Reviewer #1: Well written, a model with hypovolemic shock might have shown the benefit of the better platelet recovery.
If the clinical study has already been published (this was mentioned under Discussion, about free Hb concentration), why is the preclinical study findings being published late? Was an updated version of the machine tested in preclinical model?

Response to Reviewer #1:

The first clinical study, that was a non comparative study, was published after the first non-comparative preclinical study on swine (reference 27), using 2 models of blood loss (splenic bleeding and cardiac surgery with bypass). A preclinical version of the device (not completely assembled) was used for the first swine study and an updated version (finished designed used to obtain EU marketing authorization) for the first study in humans and the present study, although some software changes have been implemented (20w for the human study and 20y for the last animal study).

Reviewer #2: The paper “Comparative evaluation of two autotransfusion devices in a 72h survival swine model of surgically induced controlled visceral blood loss” represents an original work in its content. The paper is easy to read, with clearly defined objectives, detailed methodology. The results are clearly presented. I enjoyed reading the paper.
Good luck with the publication!

Response to Reviewer #2: Thank you for your encouragements

Reviewer #3: This study designed as a randomized controlled trial compared cell concentration and washout performances of two autotransfusion devices, a preclinical version of same™, the conventional centrifugation-based Xtra® (LivaNova, UK), with the conclusion that the same™ device showed comparable performances in terms of washout and also better performances regarding cell concentrations (RBC and platelets) without any undesirable effects.
Below are minor comments or questions:

Response to Reviewer #3: 
In the abstract you wrote : Yucatan minipigs submitted to a surgically induced controlled splenic bleeding.
In the introduction: in vivo porcine model of surgically-induced abdominal controlled hemorrhage

Thank you for the remark. We have uniformized the wording using “surgically induced controlled splenic bleeding”.

In the methods: using a previously described animal model of controlled visceral hemorrhage by surgically induced splenic lesions in minipigs [27]. Are the published results in reference 27 the same as in this manuscript?

The study described in the reference 27 is a separate study from the present report. There is no overlap of animal use nor results between both studies.

Did you perform the trials parallel? I ask, because if not, there would have to be a control for every run. Or the experiments are "mixed" in time. I suspect that the latter has happened.

The trial described in reference 27 was run prior the comparative study presented here and focused on evaluating the device performances on two model of blood loss (splenic bleeding and cardiac surgery with bypass). The experiment on the three groups in the present study were indeed mixed in time as the treatment allocation was randomized.

Can you mention the type of the used heparin please?
 Unfractionated heparin (heparin sodium 5 000 IU/mL) was used.

***Issues to do/ to consider***

226 for Xtra® device and 3 out of 1O ( please insert zero instead of Capital letter O)

Modification done and highlighted

250 same™. Cycle 2 concentrated blood in TB showed an inferior hematocrit and RBC

251 concentration for Xtra® than same™. However, median hematocrits in TB were within

252 the recommended 45 to 65% range [30] for both devices (We suggest the use the word “lower” than inferior)

Thank you for the suggestion

Please replace in the header … visceral blood loss by controlled splenic bleeding
and use in the text always controlled splenic bleeding

Thanks you for the remark, standardization of the wording using as suggested “ controlled splenic bleeding” has been implemented

Please share AABB guideline referred to in this document on recommended parameters of Auto-transfused blood as pdf as we are not able to download it online due to financial restrictions.

We are sorry that we are not able to provide the 2002 AABB guidelines as the paper book was lost during isep headquarter relocation 5 years ago. We have not been able to find another copy and the subsequent updated guidelines do not repeat the initial recommendations on the quality of the blood products. However we are providing with our submission two references that have used the same guidelines.

**Vieira SD**, da Cunha Vieira Perini F, de Sousa LCB, Buffolo E, Chaccur P, Arrais M, Jatene FB. [Autologous **blood** salvage in cardiac surgery: clinical evaluation, efficacy and levels of residual heparin.](https://pubmed.ncbi.nlm.nih.gov/31791879/) Hematol Transfus Cell Ther. 2021 Jan-Mar;43(1):1-8. doi: 10.1016/j.htct.2019.08.005.

Buys WF, Buys M, Levin AI. [Reinfusate Heparin Concentrations Produced by Two Autotransfusion Systems.](https://pubmed.ncbi.nlm.nih.gov/27591910/) J Cardiothorac Vasc Anesth. 2017 Feb;31(1):90-98. doi: 10.1053/j.jvca.2016.06.014.

Thanks again for the constructive feedback and best regards
